# A systematic review of the potential neurotoxicity of micro-and nanoplastics: the known and unknown

**DOI:** 10.1186/s12989-025-00647-4

**Published:** 2025-11-06

**Authors:** Kinga Vojnits, Andrés de León, Julien Gibon, Philip Barker, Morteza Mahmoudi, Sepideh Pakpour

**Affiliations:** 1 School of Engineering, University of British Columbia, Kelowna, BC Canada; 2 Department of Biology, University of British Columbia, Kelowna, BC Canada; 3 Office of the Vice-Principal Research and Innovation, McGill University, Montreal, QC Canada; 4 Department of Radiology & Precision Health Program, Michigan State University, East Lansing, MI USA

**Keywords:** Microplastics, Nanoplastics, Environmental pollutants, Neurotoxicity, *In vivo* models, *In vitro* models, Systematic review

## Abstract

**Background:**

The escalating accumulation of micro- and nanoplastics (MNPs) in the environment has raised significant concerns regarding their neurotoxic potential in vertebrates. This critical review synthesizes evidence from 234 original research articles across aquatic and terrestrial models, as well as in vitro systems, to evaluate the impacts of MNPs on the brain.

**Main body:**

Emerging data suggest that MNPs may reach the brain *via* olfactory translocation or by penetrating the blood–brain barrier, potentially facilitated by biomolecular corona formation. However, distribution kinetics, long-term retention, and true internal exposure levels remain unresolved. We highlight that neurotoxic outcomes, such as oxidative stress, cholinergic dysfunction, neurotransmitter imbalances, and neuronal apoptosis, vary widely depending on particle size, shape, polymer type, exposure concentration, and host species. Nevertheless, inconsistencies across models and experimental conditions, such as mismatches between oxidative stress markers and behavioral effects or lack of dose-response relationships, hinder mechanistic clarity and translational relevance to human health. Notably, most current studies employ spherical polystyrene particles at supraphysiological concentrations, limiting ecological and clinical extrapolation. Interactions with microbial biofilms and host microbiota are largely unexplored, despite their probable role in modulating neurotoxicity *via* the gut–brain axis. Moreover, most studies rely on analytical methods validated only for microplastic detection, while robust, standardized approaches for identifying nanoplastics in environmental and biological matrices remain lacking. These gaps hinder accurate exposure quantification, obscure tissue-specific accumulation patterns, and complicate human health risk estimation.

**Conclusion:**

To advance the field, we recommend comprehensive physicochemical characterization of MNPs, adoption of environmentally relevant exposure scenarios, inclusion of diverse polymer types and shapes, and mechanistic integration through multi-omics and adverse outcome pathway frameworks. Addressing these challenges through harmonized methodologies and interdisciplinary collaboration is essential for developing predictive models of MNP-induced neurotoxicity and informing human health risk assessments.

**Graphical abstract:**

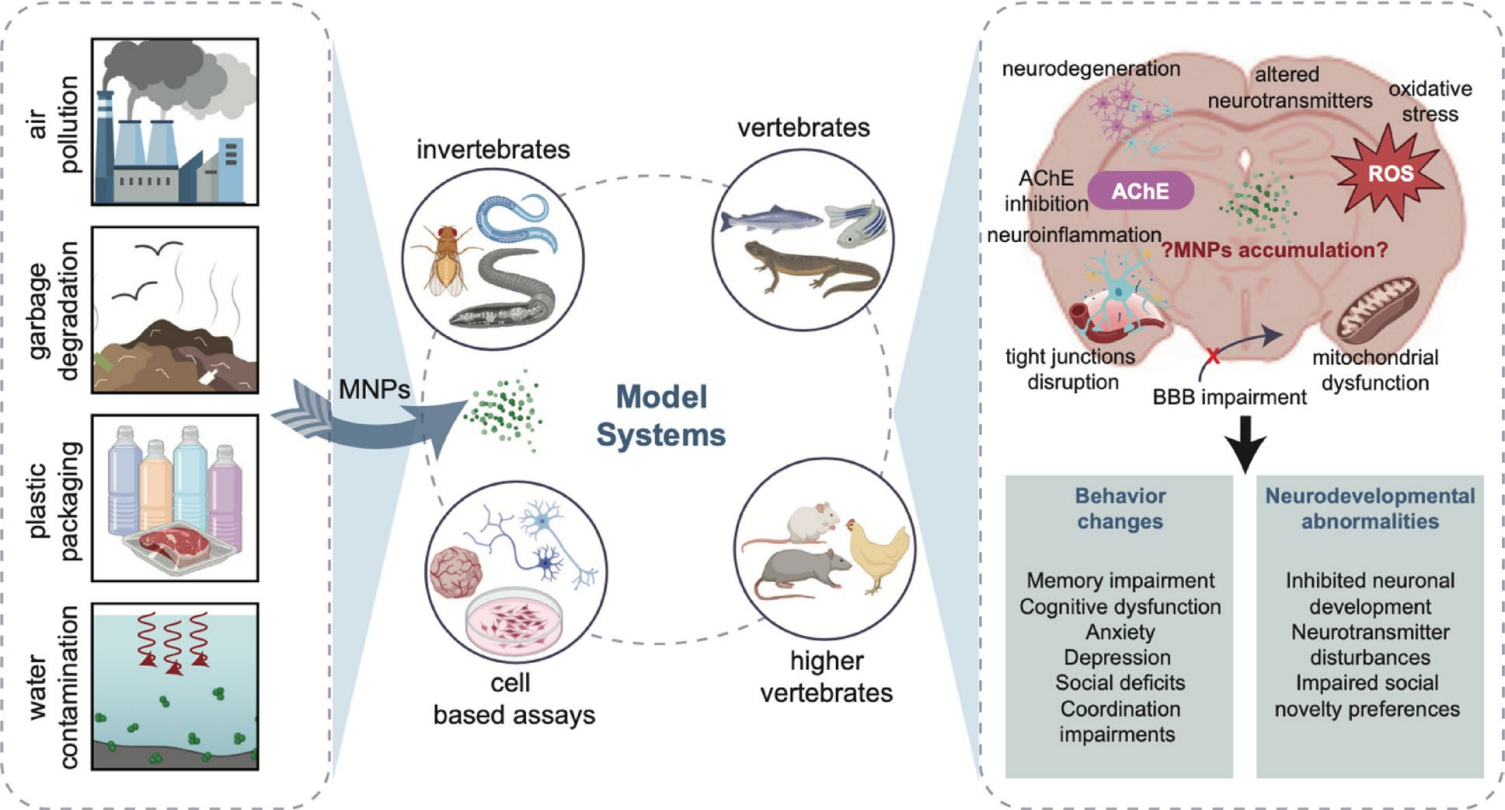

**Supplementary Information:**

The online version contains supplementary material available at 10.1186/s12989-025-00647-4.

##  Background

**Fig. 1:**
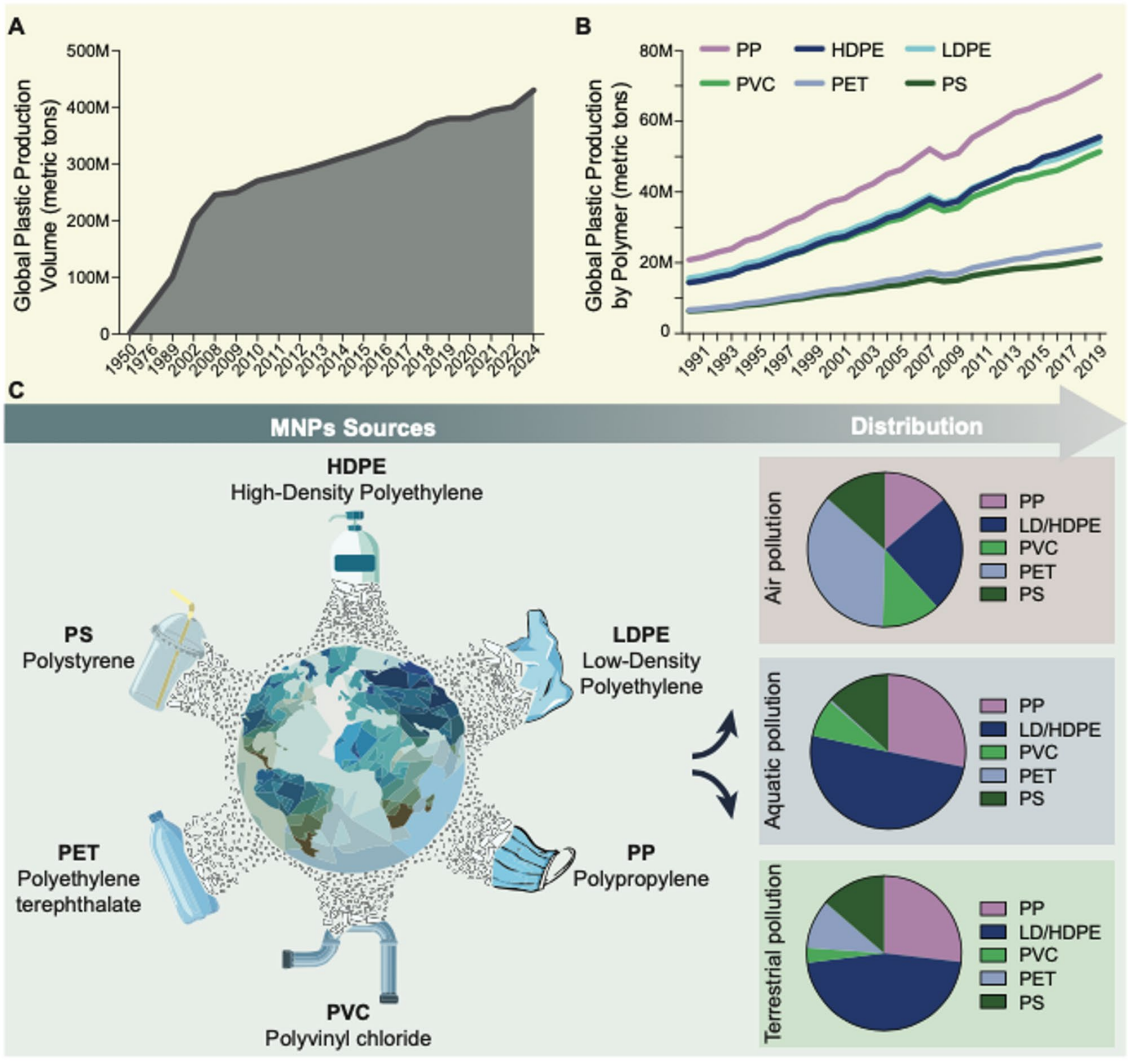
Micro- and nanoplastics (MNPs) sources and distribution in the environment. Graphs show (A) global plastic production monitored over time from 1950–2024, and (B) the increase in global plastic production by polymer from 1991–2019. (C) Sources, contamination, and environmental distribution of the most abundant MNPs types. Abbreviations: HDPE, high-density polyethylene; LDPE, low-density polyethylene; PP, polypropylene; PVC, polyvinyl chloride; PET, polyethylene terephthalate; PS, polystyrene

Plastics are among the most universally utilized materials in our society, with annual production exceeding 430 million metric tons [1, 2] (Fig. 1). Typically manufactured for single use, they result from the polymerization of various monomers or combinations of materials [3, 4].

The most abundantly produced types include high- and low-density polyethylene (HDPE and LDPE; 30%), polypropylene (PP; 18%), polyvinyl chloride (PVC; 10%), polyethylene terephthalate (PET; 9%), and polystyrene (PS; 7%) [5] (Table 1).

**Table 1:** Common utilization of the most abundant plastic types

Name	Application
Polyethylene (HDPE/LDPE)	Drink cartons, beverage cups, food and drink storage, cereal box liners, sandwich and bread bags, buckets, plastic/cling wrap, bubble wrap, grocery bags, detergent bottles, shopping bags, bin bags, laundry bags, freezer bags, toys, pipes, insulation, bottle caps, vehicle fuel tanks, protective helmets, faux-wood planks, recycled wood-plastic composites, trays, containers, work surfaces, machine parts, lids, ‘6-ring’ drink holders, protective shells, computer hardware casings, park benches, playground fixtures (slides and the like).
Polypropylene (PP)	Straws, bottle caps, prescription bottles, hot food containers, crisp bags, kettles, lunch boxes, packaging tape, disposable diapers, disposable face masks, DVD/CD boxes, clothing, surgery tools and supplies, hobbyist model.
Polyvinyl chloride (PVC)	Plumbing pipes, tubing, flooring, cladding, vinyl records, cables, cleaning solution containers, water bottles, credit cards, human and pet toys, rain gutters, teething rings, IV fluid bags, medical tubing, oxygen masks, medical containers, furniture, clothing.
Polyethylene terephthalate (PET)	Beverage bottles, cooking oil bottles, food bottles, jars (salad dressing, peanut butter, honey, etc.), packaging trays, frozen ready-meal trays, clothing, rope, first-aid blankets, polar fleece.
Polystyrene (PS)	Foam packing materials, cups, cutleries, takeout food containers, shipping and product packaging, egg cartons, building insulation.

Plastics are now ubiquitous in the environment, with an estimated 60% of all produced plastics accumulating in landfills or natural settings due to inadequate recycling and waste management [6]. Plastics are classified by size; greater than 5 mm are defined as macroplastics, less than 5 mm are microplastics (MPs), and smaller than 1 μm are referred to as nanoplastics (NPs). Micro- and nanoplastics (MNPs), comprising polymers and functional additives, are solid, insoluble particles resistant to degradation. MNPs can be directly manufactured and are added to a range of products including cosmetics and personal care products, household and industrial detergents, cleaning products, cigarette filters, paints, and fertilizers [7–10]. Approximately 145,000 metric tons of primary MNPs are used annually in the European Union [11]. MNPs can also be generated as secondary MNPs, from the laundering of synthetic fabrics, vacuum cleaning, photocopying, printing, tire erosion, and weathering and incineration of post-consumer waste [10, 12–15].

MNPs are complex pollutants with a wide variability in their physical and chemical properties, e.g., size, shape, surface area, surface charges, crystallinity, and chemical composition [16, 17]. Many associated chemicals are U.S. EPA-listed priority pollutants due to their persistence, bioaccumulation, and toxicity [18]. Moreover, MNPs can act as vectors for contaminants, including pathogens [19, 20], organic pollutants, and heavy metals [21, 22], which may alter their physical properties [21, 23] and increase complexity.

The United Nations Environment Programme recently listed MNPs among the top ten environmental concerns [24, 25], due to their presence across all habitats [26–28] and detection at every sampling location [7, 15, 29–34] (Fig. 1). While most quantification efforts focus on aquatic systems, estimating between 7,000 and 236,000 metric tons MNPs floating at sea, especially in the Mediterranean and North Pacific [13, 28, 35–37], studies on soil are limited by sampling and extraction challenges [38]. Reported soil concentrations range from 18 to 3819 particles per kg, depending on location and sampling depth [39–44]. Atmospheric MNPs concentrations range from 0.01 to 5,700 particles per m^3^, with the highest recorded in Beijing, China [33, 45–62]. Airborne MNPs are especially concerning, as they spread between terrestrial and aquatic ecosystems. Measured concentrations are probably underestimated, as current sampling techniques are less efficient at capturing nano-sized particles [63]. For instance, marine researchers typically use plankton nets (100–300 μm) and Neuston Manta nets (300–330 μm in size), which cannot capture smaller particles. Consequently, the total environmental concentration of MNPs is likely underestimated, and a recent study suggests that NPs actually comprise the dominant fraction of marine plastic pollution [63].

Humans can be directly exposed to MNPs *via* drinking water [64, 65], sea salt, air, personal care products, textiles, and indoor dust [33, 66–68], or indirectly through the food chain [69–73]. Annual intake estimates range from 70,000 to 120,000 particles, with upper limits potentially reaching 4 million, depending on lifestyle, region, and diet [74–78]. These wide ranges likely reflect differences in sampling methods, particle size detection limits, and regional exposure conditions. Once internalized, especially *via* the lungs or gut, NPs may translocate across epithelial barriers, enter the bloodstream, and disseminate systemically. Medical procedures such as intravenous therapy or blood transfusion may also introduce MNPs [79], and dermal absorption is possible under specific occupational or high-exposure conditions [80]. Consistent with these exposure routes, MNPs have been found in human lungs [81], placenta [82–84], stool [85], liver [86], kidneys [87], knee and elbow joints [88], stool [85], blood [89], and bone marrow [90]. Concerningly, MNPs have also been identified in the brain [91–93], suggesting potential risks to the central nervous system (CNS). Despite these findings and numerous reviews on human MNPs exposure [76, 80, 94–96], studies specifically addressing their effects on the brain remain limited, and mechanisms of neurotoxicity are not well understood.

Evidence from marine and freshwater studies highlights a spectrum of MNP-related impairments, including immune and neurological dysfunction, oxidative stress, and intestinal damage. Examples include red swamp crayfish [97], *Chlamys farreri* [98], and *Mytilus galloprovincialis* [99]. Additional effects have been documented in mussels [100–107], shrimps [108–110], bivalve *Corbicula fluminea* [111–114], marine mysid [115], blood clam *Tegillarca granosa* [116], wedge clam *Donax trunculus* [117], and in clam *Scrobicularia plana* [118–121]. Broad physiological disruptions have also been noted across other aquatic species [122–131]. In contrast, some studies report no neurotoxicity or oxidative stress in species such as *Dreissena polymorpha* [107], mussels [132–134], blackspot seabream *Pagellus bogaraveo* [135], *Artemia salina* [136], *Hediste diversicolor* [137], and wild sea urchins *Paracentrotus lividus* [138]. These inconsistencies underscore the complexity of MNP-induced neurotoxicity and the urgent need for standardized assessments across species.

Accurately estimating human MNP exposure is also hindered by the lack of standardized, sensitive methods capable of detecting nanoscale particles most likely to penetrate tissues [72, 139]. While pyrolysis-gas chromatography–mass spectrometry (Py-GC-MS) offers chemical specificity, it lacks resolution for particle size and count, and requires size-based separation for nanoscale detection [140, 141]. Other methods, such as inductively coupled plasma mass spectrometry (ICP-MS), flow cytometry, and Fourier-transform infrared (FTIR) or Raman spectroscopy, face limitations in resolution, reproducibility, or specificity when applied to the nanoscale range.

Finally, geographic variation, air quality, and local water infrastructure contribute to exposure variability. These limitations make it difficult to interpret toxicological data, compare risk levels, or inform public health guidance. This review, therefore, aims to synthesize current knowledge on MNP-induced neurotoxicity, explore proposed mechanisms across biological systems, and highlight gaps in exposure assessment, brain accessibility, and methodological consistency.

##  Literature search

**Fig. 2:**
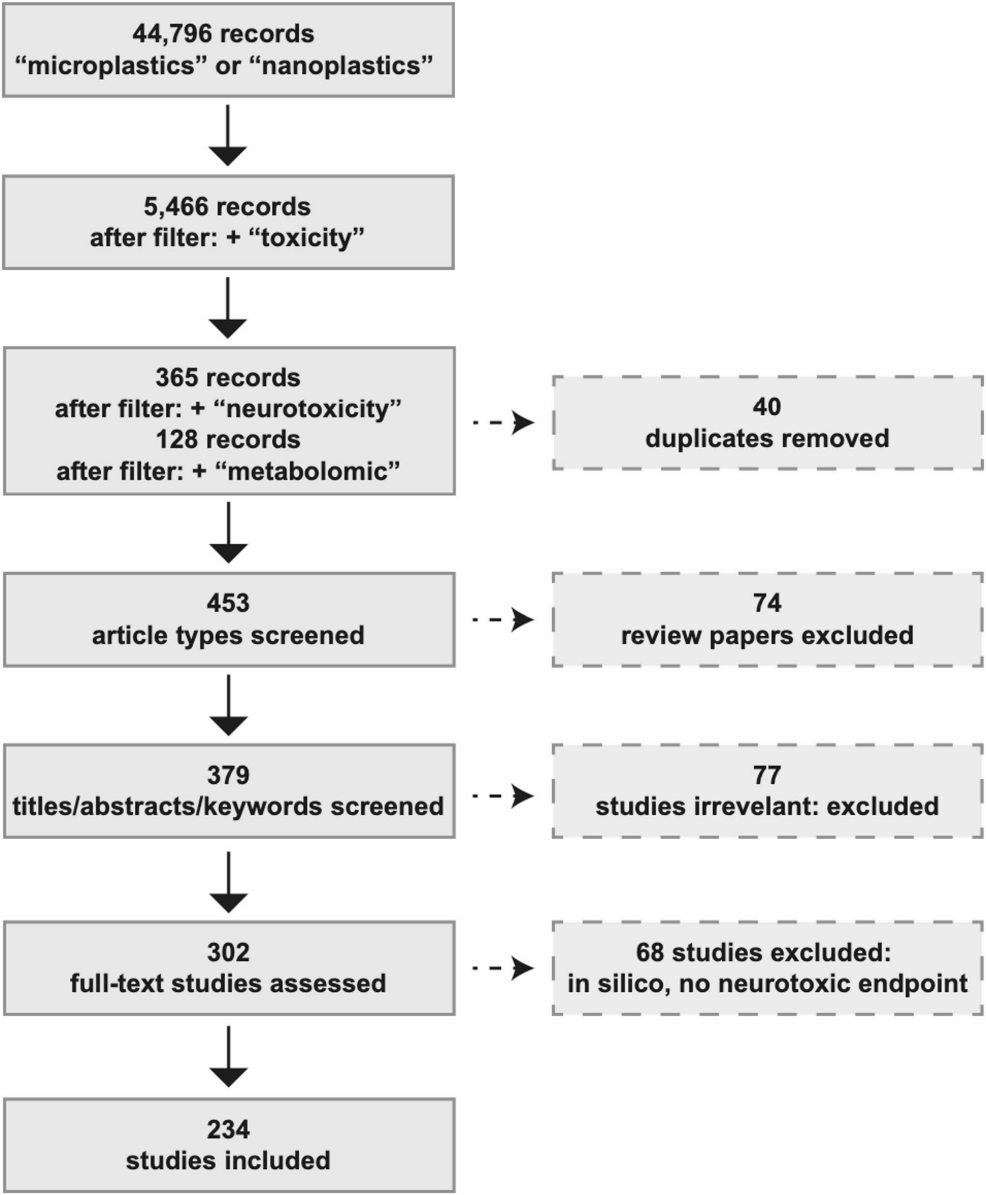
Literature search strategy for identifying studies on the neurotoxic potential of micro- and nanoplastics (MNPs). The initial search in the Medline (Ovid) database (1 January 2011 to 15 April 2025) yielded 44,796 articles using the terms “microplastics” or “nanoplastics.” This was refined to 5,466 articles when combined with “toxicity,” and further narrowed to 365 articles using the terms “microplastics” or “nanoplastics” and “neurotoxicity”, and 128 articles using the terms “microplastics” or “nanoplastics” and “metabolomic”. After excluding duplicates, reviews, in silico studies, and studies lacking neurotoxic endpoints, 234 original research articles were retained for the final analysis

To review the neurotoxic potential of MNPs, a comprehensive literature search was conducted using the Medline (Ovid) electronic database to compile relevant studies published between 1 January 2011 and 15 April 2025. The following search term combinations were used: “microplastics” or “nanoplastics” (44,796 hits), “microplastics” or “nanoplastics” and “toxicity” (5,466 hits), “microplastics” or “nanoplastics” and “neurotoxicity” (365 hits), and “microplastics” or “nanoplastics” and “metabolomic” [169]. From the 365 publications identified through the neurotoxicity-focused search and the 169 publications identified through the metabolomics-focused search, duplicates, review articles, studies that did not report explicit neurotoxic endpoints, and in silico investigations were excluded. This screening resulted in a final dataset of 234 original research articles published in English, the majority of which appeared in recent years. The literature search strategy is illustrated in Fig. 2. The final list of 234 publications is provided in Excel file format in the Supplementary Material (Supplemental Table 1). Although this scoping review adheres to the Preferred Reporting Items for Systematic Reviews and Meta-Analysis (PRISMA) guidelines, it may not be exhaustive, and studies not indexed in the Medline database could have been overlooked. However, the objective of this review is not to capture every study in this rapidly evolving field, but rather to synthesize the major themes and highlight knowledge gaps across model systems. We have distributed the selected neurotoxicity studies on MNPs across different model organisms, categorized into invertebrates (nematodes, flatworms, earthworms, worms, and flies), vertebrates (amphibians, zebrafish, and various fish species, such as *Carassius carassius*, *Carassius auratus*, *Oreochromis niloticus*, *Dicentrarchus labrax*, *Platichthys flesus*, and *Mugil cephalus*, higher vertebrates (mice, rats, and chickens), and in vitro cellular models. For publications that used both in vivo and in vitro models, each component was presented separately.

**Fig. 3:**
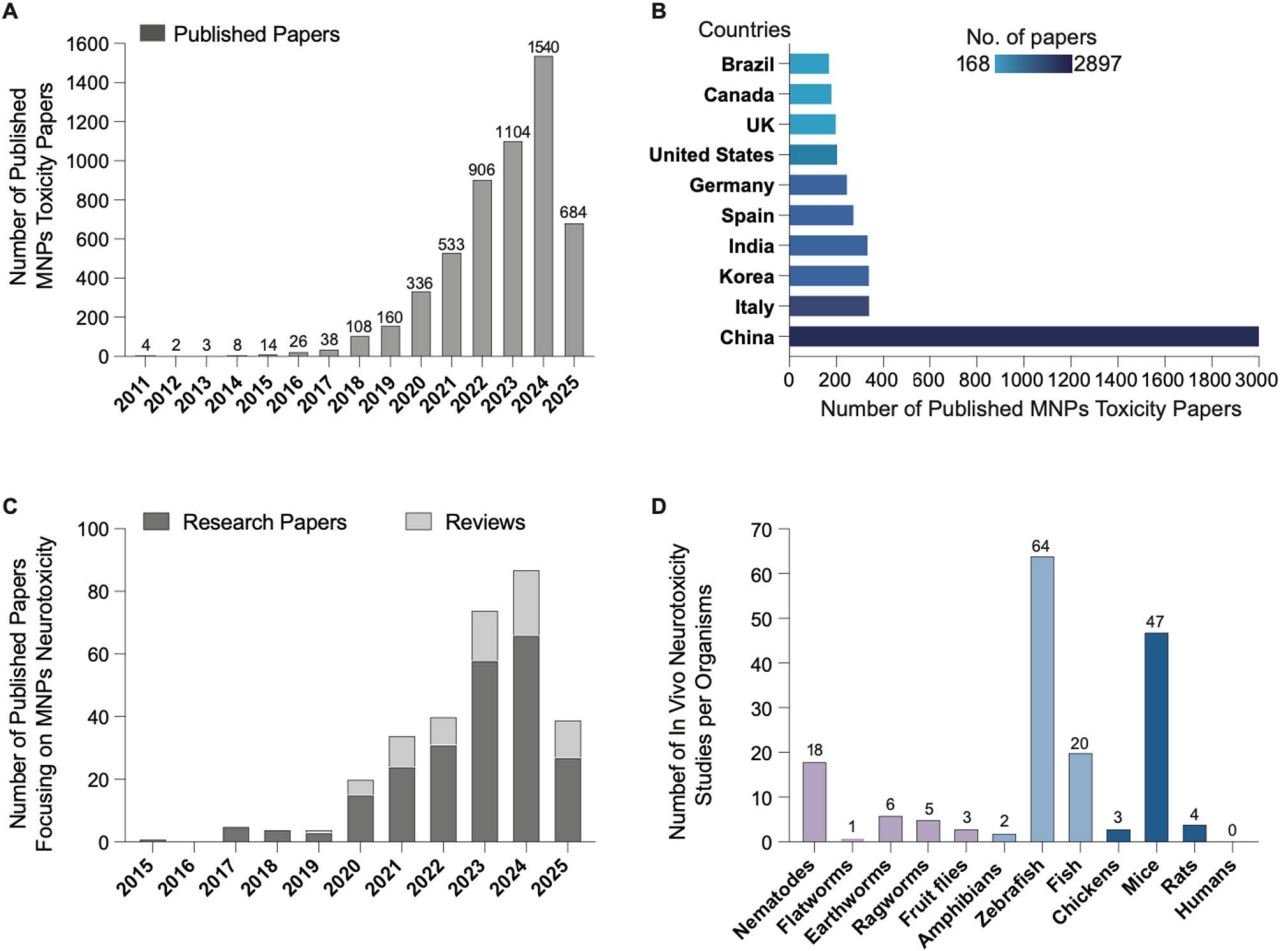
Trends in the number of publications on the neurotoxicity of micro-and nanoplastics (MNPs) over time. (A) The bar chart shows an exponential increase in published papers on MNPs toxicity from 2011 to March 2024. (B) Top 10 countries around the world that contributed to the MNPs toxicity field in terms of publications. (C) The bar chart shows the increasing number of MNPS neurotoxicity research articles and reviews published each year from 2015 to 2024, highlighting the growing interest and concern in the neurotoxic potential of MNPs. (D) Number of published in vivo studies with neurotoxicity endpoints per organism, categorized into invertebrates (light purple), vertebrates (light blue), and higher vertebrates (dark blue)

From 2011 to 2025, a total of 5,466 peer-reviewed papers specifically related to MNPs toxicity were found to meet our literature search criteria, with the annual number of publications growing exponentially over the last several years (Fig. 3A). Our analysis further revealed that China is particularly prominent in the field of MNPs’ toxicity with the assumption that the number of publications originating from a country can indicate the extent of research activity in a particular field. Collectively, the top 10 countries publishing studies on MNPs’ toxicity produced 94% of all published articles, thus representing a driving force in this research. As illustrated in Fig. 3B, China contributed 2897 articles to the total number of published articles, markedly outperforming other countries. Next in line are Italy and Korea, with 339 and 338 articles, respectively. Within the top 10 contributing countries, the articles exclusively authored by domestic researchers surpass those featuring multinational authorship, indicating that the degree of international collaboration in this research field warrants further expansion and development. With our refined search, we further identified 74 review papers and 234 original research articles specifically focusing on MNPs’ toxicity towards the nervous system (Fig. 3C). The annual number of MNPs neurotoxicity research articles and reviews published each year from 2015 to 2025 increased exponentially, highlighting the growing interest and concern in the neurotoxic potential of MNPs. Zebrafish and mice were the most frequently researched organism groups, 64 and 47, respectively (Fig. 3D).

##  Neurotoxicological consequences of MNPs exposure in invertebrates

To date, 34 studies have investigated the neurotoxic effects of PS, PE, and PET MNPs in invertebrate models, including nematodes, flatworms, earthworms, ragworms, and flies. These studies were conducted either in isolation or in combination with co-exposure to other environmental toxicants.

### MNPs neurotoxicity assessment in nematodes

#### Influence of MNP material, size, and charge on neurotoxicity and accumulation

The nematode *Caenorhabditis elegans (C. elegans)* is a well-established model for studying the adverse effects of environmental toxicants on neuronal functions due to its well-characterized genetics, short lifespan, ease of culture, and transparent body [142, 143]. Exposure to PS MNPs ranging in size from 25 nm to 1 μm, at concentrations from 0.1 to 100 µg/L, caused a range of neurotoxic outcomes, including reduced growth, impaired movement, dopaminergic neuronal damage, and altered neurotransmitter levels [144–148]. Functionalization of particles with carboxyl (-COOH) or amino (-NH_2_) groups impacted toxicity profiles, with long-term exposure to NH_2_-functionalized PS NPs showing the most pronounced effects on development, lifespan, and neuronal integrity [149–151].

#### Exposure routes, bioaccumulation, and detection

MNPs were primarily administered through ingestion in *C. elegans*, capitalizing on their natural feeding behavior. Particles were detected in the intestinal tract and associated with systemic oxidative stress and neuronal dysfunction, highlighting their potential for internal distribution.

#### MNPs as vectors of toxicants

Co-exposure studies demonstrated synergistic neurotoxicity. For example, 100 nm PE NPs combined with 6-PPD quinone (6-PPDQ), a potential neurotoxic environmental contaminant present in urban runoff and tire wear particles, led to increased neurodegeneration *via* ion channel dysregulation [152]. Combined exposure to 50 nm PS NPs and the flame retardant tetrabromobisphenol A (TBBPA) further inhibited survival, body length, and locomotor ability, while promoting reactive oxygen species (ROS) production and dopaminergic neuronal loss *via* upregulation of neurodegenerative genes such as *pink-1* and *hop-1* [153, 154].

#### Direct and indirect translocation to the nervous system

Evidence from transgenerational exposure studies shows that neurotoxicity persists beyond parental exposure. Parental treatment with 1 μm PS MNPs reduced motor activity and increased ROS in F1 progeny, suggesting indirect developmental neurotoxicity mechanisms [155].

#### Molecular and cellular toxicological impacts

Photoaged and thermally degraded MNPs (Box 1) generated environmentally persistent free radicals (EPFRs) and ROS, resulting in oxidative damage, reduced neurotransmitter levels, and altered expression of genes involved in neuronal function and development [146, 156–161].


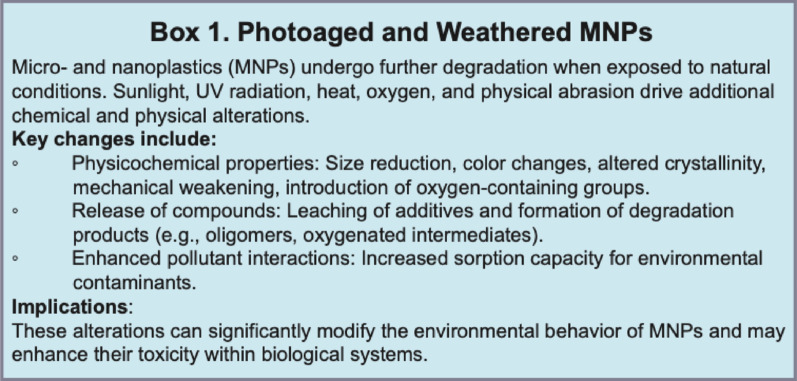


#### Behavioral and functional neurotoxicity

Behavioral deficits such as impaired locomotion, reduced heat thrashing, and altered chemotaxis responses have been consistently reported in response to MNP exposure [144–148]. Notably, long-term exposures and specific particle surface properties had more pronounced behavioral impacts. Overall, these studies indicate that neurotoxicity in *C. elegans* is influenced not only by the size and concentration of MNPs but also by particle surface properties, the occurrence of photoaging, and the presence of surface pollutants. This highlights the complex interplay between these factors and the observed toxicological effects.

### MNPs neurotoxicity assessment in flatworms, earthworms, and ragworms

#### Influence of MNP material, size, and charge impacts on neurotoxicity and accumulation

In flatworms (*Dugesia japonica*), a common freshwater species that has often been used as an indicator for the detection of aquatic environments due to their high sensitivity to environmental pollutants [162], exposure to 5 μm PS MNPs, particularly in combination with perfluorooctane sulfonate (PFOS), induced DNA damage, delayed neuronal development, and disrupted gene expression [163]. In earthworms (*Eisenia fetida*), PS and PE MNPs (200 nm–50 μm, 0.05 to 20 g/kg soil) caused neurotoxicity through inhibited acetylcholinesterase (AChE) activity, oxidative stress, and metabolic dysregulation [164–169]. Ragworms (*Hediste diversicolor*) exposed to both commercial and environmentally sourced MNPs (10–100 mg/kg sediment) exhibited size-dependent accumulation, survival reduction, and impaired antioxidant responses.

#### Exposure routes, bioaccumulation, and detection

Exposure routes in these species varied by habitat: dermal absorption and ingestion dominated in earthworms; sediment ingestion in ragworms. MNPs were shown to accumulate internally and interfere with critical detoxification and antioxidant systems [170–173].

#### MNPs as vectors of toxicants

In planarians, co-exposure with PFOS enhanced MNP-induced neurotoxicity [163]. In ragworms, comparison of MNPs and their additive dibutyl phthalate (DBP) revealed that MNPs themselves, not just additives, triggered lipid peroxidation and neuroimmune dysregulation [174].

#### Molecular and cellular toxicological impacts

In earthworms, MNP exposure impaired antioxidant enzyme activity, such as AChE, S-transferase (GST), and energy metabolism, contributing to neurotoxicity. Similarly, *H. diversicolor*, MNPs altered AChE, GST, and lactate dehydrogenase (LDH) levels, indicating disrupted neuronal and metabolic homeostasis [174].

#### Behavioral and functional neurotoxicity

Delayed neuronal development in planarians and growth and survival impairments in ragworms suggest that MNP neurotoxicity extends beyond molecular responses to whole-organism functioning, even in non-neural tissues, due to systemic oxidative stress.

### MNPs neurotoxicity assessment in flies

#### Influence of MNP material, size, and charge impacts on neurotoxicity and accumulation

The fruit fly *Drosophila melanogaster* has been used as a model organism to study human diseases, including Parkinson’s and Alzheimer’s diseases, cardiovascular diseases, and immunologic and intestinal infections, among others [175], and it is currently employed as a model in toxicology to conduct mechanistic studies on several environmental contaminants and toxicants [176]. When exposed to 2–100 μm PET MNPs at concentrations of 10–40 g/L showed significant neurotoxicity [177]. Particle size and exposure durations were key variables influencing the accumulation and severity of effects.

#### Exposure routes, bioaccumulation, and detection

PET MNPs accumulated in the midgut and hindgut following oral ingestion. The systemic impact of this accumulation was evidenced by changes in behavior and reduced physical performance in larvae and adult flies [177].

#### Molecular and cellular toxicological impacts

Though molecular-level details remain sparse in *Drosophila* studies, observed behavioral impairments point to underlying disruptions in neuromuscular coordination and neurotransmission likely mediated by oxidative damage [177].

#### Behavioral and functional neurotoxicity

Behavioral assays revealed dose- and time-dependent declines in climbing, jumping, and crawling activity [177]. These outcomes parallel findings in other invertebrate models, suggesting conserved neurotoxic effects of MNPs across species.

##  Neurotoxicity in lower vertebrates

In addition to invertebrate studies, a total of 139 studies have investigated the neurotoxic effects of MNPs in vertebrates, with a particular emphasis on aquatic species (Fig. 3D). Findings across diverse taxa consistently demonstrate MNP-induced neurotoxicity and reveal conserved disruptions in neural pathways and molecular mechanisms, suggesting common biological targets across vertebrate model systems.

### MNPs neurotoxicity assessment in amphibians

#### Influence of MNP material, size, and charge on neurotoxicity and accumulation

Amphibians such as *Rana nigromaculata* and *Xenopus tropicalis* are especially vulnerable to environmental contaminants due to their bare and permeable skin and aquatic developmental stages. These physiological traits facilitate the rapid absorption of pollutants, including MNPs [178]. Studies exposing tadpoles to PS MPs ranging from 0.1 to 10 μm, either alone or in combination with toxicants such as the antibiotic levofloxacin (LVFX), reported significant disruptions in cellular pathways associated with oxidative stress and neurotransmission [179]. Similarly, photoaged PE MNPs (36–38 μm) were shown to intensify neurotoxic effects when co-administered with triclosan, a widely used, chlorine-based antimicrobial compound [180].

#### Exposure routes, bioaccumulation, and detection

Exposure primarily occurred through immersion in contaminated water during larval development. While studies consistently reported behavioral and biochemical changes, direct detection of MNPs in brain tissue or internal organs of amphibians has remained elusive, possibly due to technical limitations in detection or rapid excretion/metabolism of the particles.

#### MNPs as vectors of toxicants

Co-exposure to MNPs and other environmental toxicants, such as LVX or triclosan, increased oxidative stress responses, which were evident from increased levels of superoxide dismutase (SOD), catalase, and altered AChE activity [180]. Interestingly, these biochemical markers did not always correlate with observed behavioral effects such as swirling movements or abnormal surface breathing, suggesting involvement of alternative mechanisms, potentially including gut microbiota-neurotransmitter interactions [181].

#### Molecular and cellular toxicological impacts

Reported effects included disruptions in cell adhesion pathways, intestinal inflammation, and alterations in gut microbiota composition. These changes are believed to influence neurotoxicity through microbiome-mediated modulation of neurotransmitter metabolism [181].

#### Behavioral and functional neurotoxicity

Exposed tadpoles exhibited lethargy, impaired feeding, and abnormal respiratory behaviors. Notably, these behavioral phenotypes were more severe with both very small and relatively large particles, indicating a non-linear, size-dependent toxicity relationship that may reflect differences in biodistribution and cellular uptake.

###  MNPs neurotoxicity assessment in fish in the environment

#### Influence of MNP material, size, and charge on neurotoxicity and accumulation

Several studies investigated the neurotoxic effects of MNP exposure in various fish species, such as crucian carp (*Carassius carassius*), goldfish (*Carassius auratus*), Nile tilapia (*Oreochromis niloticus*), red tilapia (*Oreochromis niloticus*), red drum (*Sciaenops ocellatus*), and several wild marine species, including *Dicentrarchus labrax*, *Platichthys flesus*, and *Mugil cephalus* [182–190]. Fish exposed to various polymers (PS, PE, PA) across a broad size range (nanometers to >70 μm) showed a spectrum of neurotoxic effects. Larger particles (5–70 μm) induced stronger oxidative stress, while smaller NPs were more likely to cross biological barriers and accumulate in tissues [186–199].

#### Exposure routes, bioaccumulation, and detection

In these studies, exposure primarily occurred through waterborne contamination. While the gastrointestinal tract, gills, and liver were common sites of particle accumulation, brain uptake was rarely confirmed. However, in one study involving wild *Dicentrarchus labrax*, MNPs ranging from 8 to 96 μm were detected in brain tissue, providing critical evidence for translocation across the BBB [184]. Complementing these findings, PS NPs fed to Australian bass (*Macquaria novemaculeata*) *via* trophic transfer accumulated in a size-dependent manner, with smaller particles (50 nm) localizing to brain and muscle and larger particles (1 μm) to gills and intestines [200].

#### MNPs as vectors of toxicants

MNPs have been shown to act as carriers and amplifiers for co-occurring pollutants. For example, co-exposure to PS MNPs and triphenyltin (TPT) resulted in synergistic neurotoxic effects, including altered locomotion and elevated oxidative stress. Notably, particle size affected toxicity, as 51 nm PS NPs enhanced TPT toxicity to a greater extent than 4.8 μm MPs, underscoring the increased risk of NPs exposure [201].

#### Molecular and cellular toxicological impacts

Neurotoxic responses included increased malondialdehyde (MDA), inhibited AChE activity, and observable histological changes in brain tissues [186–199]. Larger MNPs tended to induce more oxidative damage, while smaller particles primarily disrupted neurotransmitter function and synaptic activity [202].

#### Behavioral and functional neurotoxicity

Exposed fish displayed a range of behavioral alterations, such as impaired olfactory response and abnormal locomotion. In red tilapia, 5 μm PS MNPs specifically inhibited brain AChE activity, while exposure to smaller NPs produced more subtle but widespread neurophysiological disruptions [202]. These findings support the role of particle size and surface characteristics in determining the severity and nature of neurobehavioral toxicity.

### MNPs neurotoxicity assessment in zebrafish lab models

#### Influence of MNP material, size, and charge on neurotoxicity and accumulation

Over the last few decades, the zebrafish, *Danio rerio*, has emerged as a prominent vertebrate model in biomedical and environmental research due to its high genetic homology with humans [203], transparent embryos, ease of genetic manipulation, rapid development, and low maintenance cost. These traits, along with their high productivity and suitability for high-throughput screening, have made zebrafish widely used for disease modeling [204–207], drug development [206, 208, 209], and especially for toxicological studies [210–212], including investigations into the neurotoxic effects of MNPs [213, 214].

Numerous studies have examined the size- and charge-dependent toxicity of MNPs in zebrafish, testing polymers such as PS, PE, and polylactic acid (PLA) in a wide size range from 20 nm to over 50 μm [203, 215–221]. Results consistently showed that smaller particles (20–100 nm) caused more severe neurotoxicity due to their enhanced ability to cross biological barriers and accumulate in brain tissues [222–225]. Particle surface charge also significantly influenced toxicity; positively charged MNPs (e.g., amine-functionalized PS MNPs) induced greater apoptosis, oxidative stress, and behavioral abnormalities than neutral or negatively charged ones [226–228].

#### Exposure routes, bioaccumulation, and detection

Zebrafish have been exposed to MNPs through various routes, including waterborne exposure, dietary intake, and direct microinjection. In one comparative study, zebrafish exposed to PE MNPs (40–47 μm at concentrations of 0.1–10 mg/L) through both waterborne and foodborne routes exhibited distinct patterns of neurotoxicity. Although waterborne exposure led to higher ingestion rates, foodborne exposure caused more severe sublethal effects, such as abnormal hyperactivity and disrupted swimming behavior [229]. Neurochemical analysis revealed that the foodborne route predominantly affected the dopaminergic system, while the cholinergic pathway was more disrupted in the waterborne group, suggesting route-specific mechanisms of neurotoxicity [229].

MNP accumulation has been observed in multiple tissues, including the brain, liver, gills, gut, and eyes. Notably, smaller particles were capable of crossing the BBB and the chorion in developing embryos [222–225].

#### MNPs as vectors of toxicants

Zebrafish co-exposed to MNPs and environmental toxicants, such as arsenic, polybrominated diphenyl ethers (PBDE-47), nonylphenol (4-NP), fluoxetine, and copper, displayed increased oxidative stress, neuroinflammation, and disruption of neurotransmitter systems [229–243]. MNPs facilitated the bioaccumulation of these toxicants, especially in neural tissue, and altered the microbiota-gut-brain axis, leading to serotonin pathway disruption and depressive-like behaviors [244–246].

For instance, exposure to aged 1 μm PS MNPs in combination with the insecticide thiamethoxam reduced heart rate and locomotor activity in larvae, suppressed antioxidant enzyme function, and disturbed neurotransmitter homeostasis [247]. Similarly, MNPs have been shown to carry methylmercury (MeHg), a potent neurotoxicant [248–250], contributing to elevated oxidative stress and motor deficits in zebrafish [251, 252].

Other studies demonstrated synergistic effects of cosmetic ingredients such as avobenzone (AVO) co-occurring with MNPs in aquatic environments. This mixture impaired AChE activity, reduced antioxidant defenses, and altered retinal and neural development in zebrafish larvae [253]. Moreover, experiments combining different PS NPs (e.g., COOH-, NH_2_-functionalized, and unmodified PS NPs) with acrylamide, a well-known neurotoxicant [254], revealed that 100 nm PS NPs-COOH most significantly exacerbated neurodevelopmental toxicity, increased embryo mortality, and elevated heart rate [255].

Exposure to MNPs mixed with the pesticide abamectin also resulted in hepatic and renal damage, inhibited catalase and acid phosphatase activity, but did not affect glutathione S-transferase or AChE levels [256]. A field-based study further evaluated the real-world implications of MNP-mediated pollutant transfer. MNPs composed of PE, PP, PS, and PVC were aged on the Rhine River surface for 21 days. While zebrafish exposed to water samples exhibited notable behavioral changes and altered cyclophilin 40 (CYP40) and AChE activity, the effects of clean or pollutant-loaded MNPs were considerably milder [257]. These results suggest that under natural conditions, MNPs may have limited capacity for transferring pollutants to aquatic organisms compared to naturally suspended matter.

#### Direct and indirect translocation to the brain

Brain accumulation of MNPs has been confirmed through fluorescence imaging and electron microscopy [222, 223]. Smaller 20 nm PS NPs demonstrated higher translocation efficiency across the BBB and induced more severe neurobehavioral outcomes compared to 100 nm–1 μm particles [222–225]. This supports a particle size-dependent model of neuroinvasion and toxicity.

#### Molecular and cellular toxicological impacts

MNP exposure in zebrafish has been linked to a range of neurotoxic responses, including neuroinflammation, apoptosis, altered expression of neurotransmitter-related genes (e.g., GABAergic, cholinergic, serotonergic pathways), and microglia/astrocyte activation in CNS tissues [216, 258, 259]. These effects were more often severe following exposure to photoaged MNPs, which are more chemically reactive due to surface oxidation [260–264]. Charge- and size-dependent cellular effects were repeatedly observed [226–228, 265–267], underscoring the complex interaction between particle characteristics and toxicodynamic profiles. Furthermore, in elderly zebrafish, PS MP exposure induced dose-dependent hepatic oxidative stress, immune activation, and metabolic disruptions, especially in pathways linked to energy, immunity, and neurological function, suggesting systemic mechanisms that may exacerbate MNP-associated neurotoxicity [268].

#### Region-specific CNS impacts

Morphological abnormalities were reported in specific brain regions such as the optic tectum and hindbrain, including disrupted neural differentiation and impaired synaptic development [269, 270]. Organ-specific disruption patterns varied depending on MNP size and polymer type [271–274], suggesting selective vulnerability of certain CNS regions.

#### Behavioral impairments and sexual dimorphism

Zebrafish exhibited a wide range of behavioral deficits following MNP exposure, including reduced locomotion, anxiety-like behavior, circadian rhythm disruption, and learning and memory impairments [275]. Notably, sex-specific differences were observed in co-exposures with endocrine-disrupting chemicals like 4-methylbenzylidene camphor (4-MBC) or flame retardants. Females showed autism spectrum disorder (ASD)-like behavior and impaired oocyte development, while males displayed Parkinson’s-like motor symptoms and impaired spermatogenesis [276, 277].

#### Relevance to human disease

Many MNP-induced phenotypes observed in zebrafish, such as altered neurotransmission, oxidative stress, and neurodevelopmental disruption, mirror features of human neuropsychiatric conditions, including depression, Parkinson’s disease, ASD, and schizophrenia [278]. Studies using schizophrenia zebrafish models showed that MNP exposure exacerbated neural lesions and increased oxidative damage, reinforcing the model’s translational value for studying environmental contributors to human brain diseases.

##  MNPs neurotoxicity assessment in higher vertebrates

Compared to invertebrates and lower vertebrates, there is a notable scarcity of research using higher vertebrates to investigate MNP neurotoxicity. However, this area of research is rapidly gaining attention. Notably, 23 of the 55 identified studies in this category were published within the past year (Fig. 3C, Supplemental Table 2), reflecting growing concern about MNP-related risks to mammalian and avian nervous systems.

### Neurotoxicity effects of MNPs in mouse models

#### Influence of MNP material, size, and charge on neurotoxicity and accumulation

Studies using mouse models have primarily focused on PS NPs (25–400 nm) and MPs (up to 10 μm), administered at doses ranging from 1 mg to 50 mg *via* oral gavage. Behavioral outcomes included anxiety-like behavior, depression, social deficits, and, in some cases, impaired spatial learning and memory, though not all studies found consistent effects on cognition [279–285]. Cognitive decline and memory impairments have been documented following exposure to both nano- (50 nm) and micro-sized (5–10 μm) PS particles [286, 287], with toxicity severity depending on size, dose, and surface chemistry [288].

Specific studies found that 30–50 nm PS NPs administered at 10–20 mg/kg/day impaired spatial and fear memory without altering locomotion or social behavior [289]. In contrast, other polymers have been less frequently studied. PP MNPs (7–20 μm), with or without Di-(2-Ethylhexyl)Phthalate (DEHP), administered at 5 mg/kg, induced hippocampal CA3 damage and cognitive deficits [290]. PET NPs (83 nm, 200 mg/kg) triggered neuroinflammation and oxidative stress [291], and oxidized and irregularly shaped LDPE MNPs (2.67–12.61 μm) disrupted cholinergic signaling more than unoxidized forms [292]. Moreover, PLA oligomers were shown to accumulate in the midbrain and cause mitochondrial calcium overload, leading to Parkinson’s-like neurotoxicity [293].

#### Exposure routes, bioaccumulation, and detection

Oral gavage remains the most common exposure method, although inhalation and intranasal routes have also been employed. Intranasal administration of amine-functionalized PS-NH_2_ NPs resulted in greater brain accumulation compared to carboxylated (PS-COOH) or unmodified particles and led to NF-κB activation and impaired glymphatic dysfunction [294]. Ingested PS MPs have similarly been detected in distant tissues, including the brain, liver, and kidney, with concentration- and particle type-dependent metabolic alterations in the colon, liver, and brain, further supporting systemic distribution and potential CNS involvement [295]. Inhalation studies have demonstrated translocation to multiple brain regions, including the olfactory bulb, cerebrum, cerebellum, and pons, especially for PS-NH_2_ particles, though particle detection in tissues is not consistently reported across studies [279, 280, 296–313].

#### MNPs as vectors of toxicants

MNPs have been shown to enhance the toxicity of environmental co-contaminants. For instance, co-exposure with DEHP, a widely present endocrine disruptor in soil, air, water, and food [314], resulted in synergistic effects, including cognitive dysfunction, oxidative stress, and gut-brain axis disruption [315–317]. These findings align with epidemiological links between DEPH and increased risk for anxiety and autism in children [314, 318].

Similar results were observed when MNPs were co-exposed with iron [317], silver nanoparticles [319], or antibiotics such as doxycycline. Combined exposure to 500 nm PS MNPs and doxycycline altered gut microbiota homeostasis, increased brain inflammation, and impaired learning and memory [320]. Importantly, fecal microbiota transplantation reversed many of these deficits, underscoring the role of the microbiota-gut-brain axis. However, further studies are needed to understand the dynamics of co-exposure uptake, bioaccumulation, and metabolism.

#### Direct and indirect brain translocation

Several studies confirmed MNP accumulation in the brain through imaging or tissue analysis [307, 308, 311, 321], while others observed CNS dysfunction in the absence of detectable particles, suggesting indirect mechanisms, such as systemic inflammation or gut-brain communication [310]. Maternal exposure to PS MNPs (50 and 500 nm) during gestation and lactation induced sex-specific neurodevelopmental abnormalities and long-term cognitive deficits in offspring [322].

#### Molecular and cellular toxicological impacts

Across studies, MNP exposure was associated with neuroinflammation, microglial activation, dendritic spine loss, altered mitochondrial function, decreased AChE activity, and apoptosis [279, 280, 296–310]. Exposure also reduced BBB integrity *via* suppression of tight junction proteins, such as zonula occludens-1 (ZO-1) and occludin. Short-term exposure to PS MPs further induced behavioral alterations alongside immune marker changes in brain tissue, with age-dependent differences suggesting heightened vulnerability during both early-life development and aging [323].

#### CNS cell and regional impacts

Neurodegeneration was frequently observed in hippocampal neurons and cerebellar Purkinje cells, along with inhibition of neurogenesis and activation of glial cells [279, 280, 309]. Particle accumulation showed regional specificity, particularly in the olfactory tubercle and cerebellum [308].

#### Behavioral impairments and sexual dimorphism

Behavioral phenotypes included anxiety-like behaviors, social impairments, and cognitive deficits. Several studies highlighted sex-specific effects, with female mice showing greater oxidative stress and behavioral changes [322, 324], potentially due to interference with estrogen signaling and brain sexual differentiation.

#### Relevance to human diseases

Neurotoxic pathways activated by MNPs, such as ferroptosis, inflammation, mitochondrial dysfunction, and BBB disruption, overlap with the pathological features of human neurological diseases, including Parkinson’s disease, Alzheimer’s disease, and ASD.

### Neurotoxicity effects of MNPs in rats

#### Influence of MNP material, size, and charge on neurotoxicity and accumulation

Studies in rats have primarily used PS MNPs (17 nm and up), though data on particle accumulation are often lacking, making mechanistic interpretation more challenging [325–327]. Nevertheless, consistent findings suggest potential neurotoxicity, especially following developmental exposure.

#### Exposure routes, bioaccumulation, and detection

In these studies, most exposures involved oral gavage or maternal administration during gestation and lactation. Brain accumulation of MNPs was rarely measured directly. However, offspring from exposed dams exhibited cognitive impairment and hippocampal injury, indicating developmental and potentially transgenerational CNS effects [327].

#### Direct and indirect brain translocation

Although brain uptake was generally not confirmed, indirect evidence from maternal exposure models, such as hippocampal damage and cognitive impairment in offspring, points to neurodevelopmental vulnerability during gestation [327].

#### Molecular and cellular toxicological impacts

Mechanistic studies identified elevated ROS production, ferroptosis, and P53-mediated ferritinophagy in brain tissues [327]. Altered astrocyte proportions and reduced antioxidant enzyme activity (e.g., GST, peroxidases) were observed, with more pronounced effects in females [325], likely involving disrupted estrogen pathways [326]. In contrast, a recent study of early-life oral exposure to nylon-11 or PS NPs in rat pups found no changes in brain neurotransmitter levels or related metabolites. However, plasma metabolomic alterations in amino acid and lipid pathways suggest systemic metabolic disruption that may indirectly affect neurodevelopment [328].

#### Behavioral impairments and sexual dimorphism

Behavioral assays revealed significant sex-specific effects, including differences in grooming behavior, stress responses, and fecal boli counts. These outcomes suggest altered neuroendocrine regulation and stress reactivity [326].

#### Relevance to human disease

Neurobiological pathways disrupted by MNPs in rats, particularly those involving oxidative stress, hippocampal plasticity, and ferroptosis, mirror mechanisms implicated in human developmental neurodegenerative diseases.

### Neurotoxicity effects of MNPs in chickens

#### Influence of MNP material, size, and charge on neurotoxicity and accumulation

Studies in chickens employed PS MNPs ranging from 500 nm to 50 μm, typically administered *via* drinking water or oral gavage. MNP accumulation was confirmed in peripheral tissues (e.g., liver, intestine, muscle), but not in the brain [329–331].

#### Exposure routes, bioaccumulation, and indirect CNS impacts

Despite the lack of detected brain accumulation, systemic MNP exposure produced significant neurotoxic effects, likely mediated by peripheral inflammation, oxidative stress, or metabolic disruptions.

#### Molecular and cellular toxicological impacts

Neurotoxicity markers included reduced AChE activity, oxidative stress, inflammatory cytokine release, altered lipid metabolism, and loss of BBB integrity. Histopathological signs such as cerebral hemorrhage and microthrombi formation were also reported, despite no direct particle detection in brain tissue [329–331].

#### Behavioral and functional neurotoxicity

Although behavioral outcomes were not directly assessed, histological damage, such as Purkinje cell loss and vascular injury, strongly suggests CNS dysfunction.

#### Relevance to human disease

These findings provide additional evidence that neurotoxicity may occur *via* indirect pathways without direct CNS accumulation of MNPs. They emphasize the potential roles of systemic inflammation, barrier disruption, and microbiota in mediating MNP-related neuropathology.

### Gaps in the current higher vertebrate models testing neurotoxicity of MNPs

**Fig. 4:**
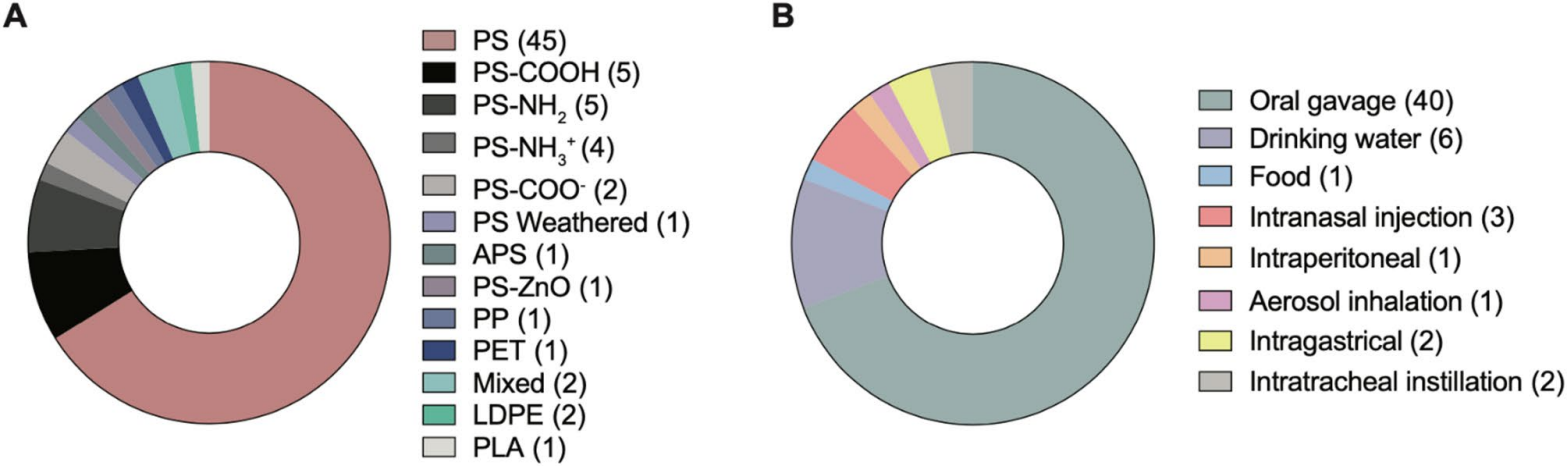
The most common polymers and administration routes applied in higher vertebrate studies. (A) Type of micro- and nanoplastics (MNPs) beads used in the higher vertebrate studies. (B) Different administration routes of MNPs exposure studies in higher vertebrates

Despite the growing interest and models assessing MNP neurotoxicity, research remains largely focused on specific polymer types, particularly in higher vertebrate models. The most common polymers produced globally, including PE, PP, PVC, PET, and PS, often enter the environment as heterogeneous plastic mixtures. Environmental fragmentation and degradation further complicate their classification, generating products with different shapes, sizes, chemical compositions, and densities. However, all higher vertebrate animal model studies have primarily focused on PS bead particles (45 studies), except for one-one study that assessed the effect of PP, PET, LDPE, and PLA bead exposure, with none investigating known heterogeneous mixtures found in the environment (Fig. 4A, Supplemental Table 2). Moreover, MNPs exhibit diverse shapes and surfaces from environmental weathering that may influence biodistribution and their adverse effects. To this end, surface-modified, i.e., carboxylated, amino-modified, and weathered PS MNPs should be assessed and compared to virgin PS MNPs. Despite the characterization of exposure effects of a particular polymer, with positively or negatively charged functional groups, or with surface modifications, commercial specifications do not reflect environmental exposure accurately [139, 332].

The environmentally relevant dose of MNPs exposure is heavily debated. Many experimental studies apply unrealistic high concentrations of MNPs, 0.75–3.0 × 10^5^ particles per cm^3^ ± 20%, which is far greater than current human exposure estimates [333]. In the above-described experiments (Supplemental Table 2), concentrations ranged from 1 µg/L to 250 mg/L without information on particle density; thus, the exact exposure number of particles is unknown. Therefore, it is critical to determine the threshold at which MNPs exposure is associated with adverse events.

Oral ingestion and drinking water administration have been the focus of MNPs research to date (Fig. 4B, Supplemental Table 2). However, both exposure routes have limitations. Low absorption and particle size limitations (up to 20 μm) were reported with oral ingestion. With drinking water administration, the main limitations are unknown particle intake calculations, inefficient for low-density polymers, such as PP and PE, considering particle sedimentation over time, and bioavailability, which was estimated to range from 0.2 to 1.7% with different types of MNPs in vivo [334]. MNPs are not only absorbed by food or water through the digestive tract, but they can also be absorbed by skin or inhaled through fine dust in the air. Therefore, other exposure routes should also be considered, especially in line with previous studies in zebrafish showing that bioaccumulation and adverse effects might depend on the exposure routes and uptake of MNPs.

**Fig. 5:**
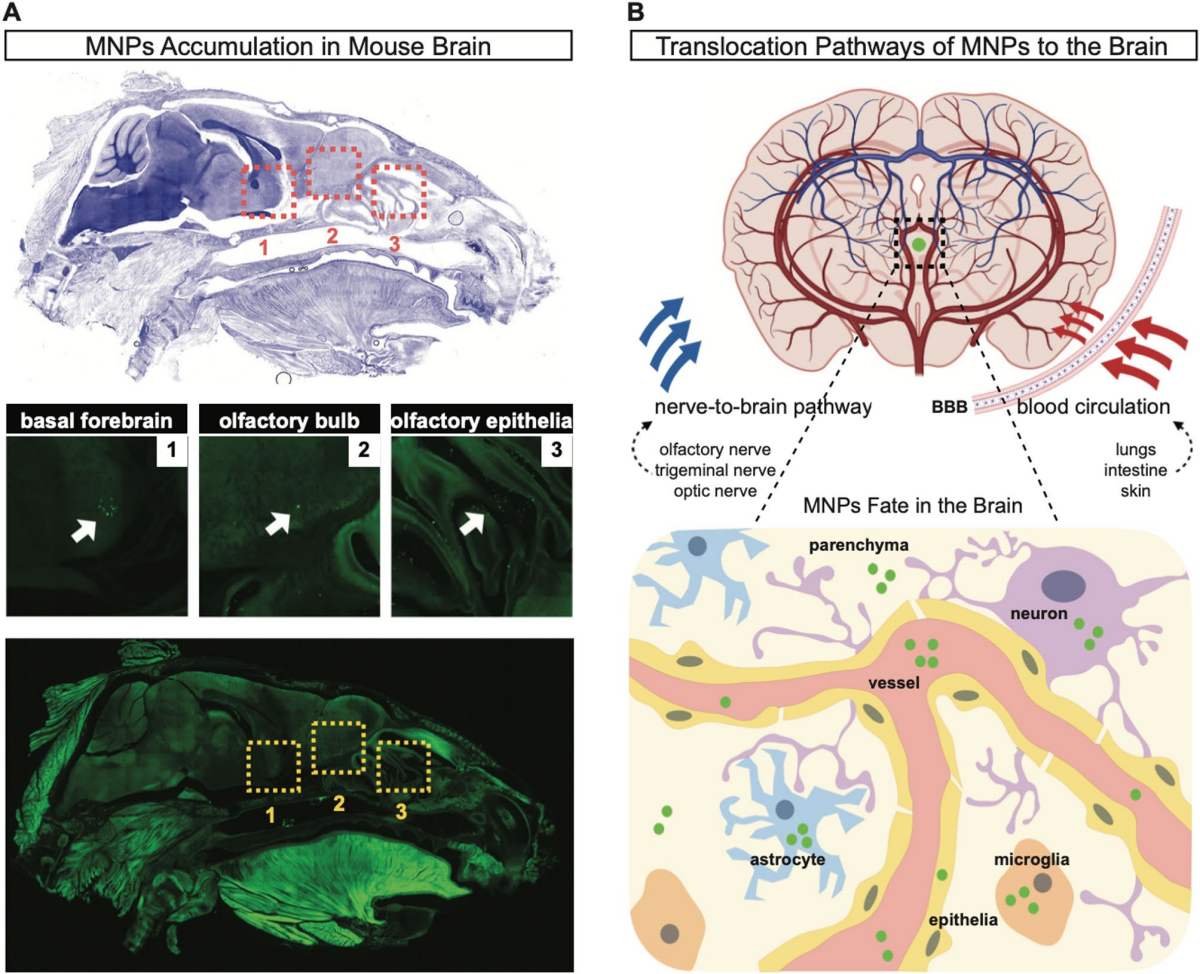
Micro- and nanoplastics (MNPs) accumulation and translocation pathways to the brain. (A) Representative fluorescence microscopy image of a sagittal section of a mouse head following intranasal administration of 2 μm green-fluorescent polystyrene microplastics (PS MPs). MPs are detected in the dashed square areas, indicating their presence in the olfactory epithelia, olfactory bulb, and basal forebrain. White arrows mark the locations of the MPs [321]. Top panel: DAPI-stained sagittal section of the mouse head. Bottom panel: same sagittal section of the mouse head scanned at an excitation wavelength of 488 nm to detect the presence of MPs. (B) MNPs can reach the brain through various routes, including direct access *via* the olfactory epithelia, which are in close contact with the olfactory bulb, the trigeminal nerve, optic nerve, or through the vascular system, where MNPs may be transported from other entry points such as the gut, lungs, or skin. Although MNPs have been detected in the brain, their ultimate destination within the organ remains unclear. These particles could either remain confined to the vascular system or cross the blood-brain barrier (BBB) epithelium. Once in the brain parenchyma, MNPs may stay free or infiltrate different cell types, including neurons, astrocytes, and microglia. Further research is necessary to better understand their fate within the brain. Abbreviation: BBB, blood-brain barrier

The brain is one of the most vulnerable organs in any animal organism, and it is generally considered to be protected by the BBB from exogenous compounds in the circulation. However, various nanoparticles can overcome this physical barrier and enter the brain through mechanisms that have yet to be elucidated [335]. So far, two different pathways have been proposed [335, 336]. One pathway for nanoparticles, i.e., carbon, gold, and manganese dioxide, to reach the brain is by sensory nerve endings in airway epithelia or the olfactory bulb, followed by axonal translocation to brain structures [337–339]. Another pathway is the uptake through the BBB *via* systemic distribution [340–342]. Whether MNPs, similar to nanoparticles, can enter or accumulate in the mammalian brain remains a subject of debate. In fish, MNPs can cross the BBB in a size-dependent manner [343]. In contrast, to our knowledge so far, only four rodent studies showed that 17–100 nm and 2 μm PS MNPs could reach brain tissues [289, 297, 321, 326], and one study revealed the role of biomolecular corona formation for NPs passing the BBB [344]. The extent to which MNPs can breach the BBB and accumulate in the brain parenchyma, where neurons are directly exposed, has yet to be investigated (Fig. 5).

##  In vitro neurotoxicity

**Fig. 6:**
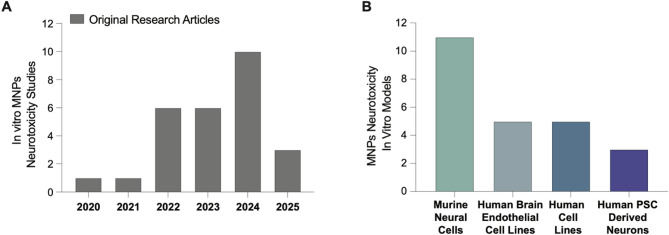
Trends in the number of in vitro studies on the neurotoxicity of micro-and nanoplastics (MNPs) over time. (A) The bar chart shows a rapid increase in published papers on in vitro MNPs neurotoxicity from 2020 to 2024. (B) Number of published in vitro studies with neurotoxicity endpoints per cell type

Animal models remain the gold standard in preclinical research; however, applying in vitro assays for hazard assessment of MNP exposure offers advantages in throughput and mechanistic insight. Despite rapid expansion in the field, relatively few neural cell-based in vitro studies have investigated MNP-induced neurotoxicity to date (Fig. 6).

### Neurotoxicity on murine primary neural cells and cell lines

#### Influence of MNP material, size, and charge on neurotoxicity and accumulation

The neurotoxic potential of MNPs in murine neural models varies significantly with particle size, surface chemistry, and charge. Several studies have confirmed that smaller particles and positively charged surface modifications enhance cellular uptake and cytotoxicity. For instance, 25–50 nm PS MNPs were taken up by primary rat microglia and astrocytes, leading to concentration-dependent cytotoxicity and microglial activation, as evidenced by morphological changes and increased immunostaining of activation markers [289, 345].

Similarly, 80 nm PS-NH₂ particles were preferentially internalized in rat primary neurons compared to neutral or negatively charged variants, suggesting that surface charge is a key determinant of neuronal MNP accumulation [312]. Studies in rat and mouse neural stem cell (NSC)-derived cultures showed that astrocytes and NSCs internalized smaller MNPs more readily, with positively charged PS MNPs reducing cell viability and proliferation [346, 347]. Notably, 2 μm PS particles were too large to enter the plasma membrane of neurons but still induced significant neurotoxicity, such as dendritic shortening and apoptosis, through indirect mechanisms [324].

#### Exposure routes, bioaccumulation, and detection

Most in vitro models use static exposure conditions, which may not fully capture the dynamics of particle-tissue interactions. However, experiments using HT-22 hippocampal cells in microfluidic (flow-based) devices demonstrated that dynamic exposure to 100 nm and 1 μm PS MNPs (5–75 µg/mL) led to greater cytotoxicity, increased ROS production, and cell cycle arrest compared to static cultures [348]. This suggests that more realistic exposure models are critical to accurately assess MNP neurotoxicity.

#### MNPs as vectors of toxicants

Although this role was not directly tested in murine in vitro systems, the physicochemical traits that enhance MNP uptake, i.e., small size and surface reactivity, could also facilitate the adsorption and transport of co-contaminants. This implies that MNPs may act as vectors that amplify the neurotoxic effects of environmental toxicants.

#### Molecular and cellular toxicological impacts

Studies using cell lines like PC12 and SH-SY5Y, as well as mouse cortical neurons, consistently report cytoskeletal alterations, dendritic atrophy, cell viability loss, and apoptotic cell death following exposure to 18 nm sulfonated PS MNPs [348]. These effects were both time- and dose-dependent. Key molecular markers included caspase-3 activation and loss of structural integrity (e.g., soma and dendritic shortening) [349–351]. Additionally, signs of reactive astrogliosis and reduced oligodendrocyte markers suggest broader dysregulation of CNS glial cell function.

#### CNS cell and regional impacts

Different neural cell types exhibited varying susceptibility to MNPs. Microglia and astrocytes, due to their phagocytic and immune-responsive nature, appear to internalize MNPs more efficiently and respond with elevated pro-inflammatory signaling [346]. NSCs are particularly sensitive to disruption of proliferation and lineage specification, highlighting developmental vulnerability. Neurons, while less efficient at MNP uptake, appear to be affected indirectly through glia-mediated inflammation and oxidative stress.

#### Behavioral impairments and sexual dimorphism

Although in vitro systems cannot model complex behaviors, the cellular and molecular disruptions observed, especially oxidative stress, neuroinflammation, and apoptosis, are mechanistically linked to cognitive dysfunction and neurodegenerative conditions in vivo. Sexual dimorphism remains largely unexplored in these models; future studies using sex-specific primary cultures or hormone-supplemented media could address this critical gap.

#### Relevance to human disease

Molecular changes such as caspase activation, mitochondrial dysfunction, and chronic inflammation mirror key features of human neurodegenerative disorders, including Alzheimer’s and Parkinson’s diseases. These findings strengthen the translational relevance of murine in vitro studies for understanding human pathophysiology linked to environmental MNP exposure.

### Neurotoxicity on human brain endothelial cell lines

#### Influence of MNP material, size, and charge on neurotoxicity and accumulation

The brain endothelial phenotype of the hCMEC/D3 human brain microvascular endothelial cell line has been extensively characterized and is a widely used model of BBB function. Exposure to 100 nm PS MNPs (30 µg/mL) for 24 h did not significantly decrease cell viability; however, significant decreases in the expression of tight junction proteins ZO-1 and occludin were observed, particularly in cells exposed to PS-COOH and PS-NH₂ MNPs, suggesting subtle barrier compromise even in the absence of overt cytotoxicity [279].

#### Exposure routes, bioaccumulation, and detection

Although no internalized MNPs were detected in hCMEC/D3 cells in this study, previous work using larger (0.2–2 μm) PS particles reported internalization and consequent inflammatory signaling, including ROS production and NF-κB activation [302, 352]. The discrepancy may reflect differences in particle size, surface chemistry, detection methods, or culture conditions.

#### Molecular and cellular toxicological impacts

PS MNP exposure altered the expression of junctional and matrix-modifying proteins, including upregulation of matrix metalloproteinase-9 (MMP-9), suggesting possible extracellular matrix remodeling and compromised BBB integrity. However, the lack of trans-endothelial electrical resistance (TEER) measurements limits definitive conclusions regarding barrier permeability.

#### Relevance to human disease

BBB disruption is a key feature in many CNS disorders, including multiple sclerosis, epilepsy, and Alzheimer’s disease. The observed protein expression changes suggest that chronic MNP exposure could contribute to neuroinflammation *via* BBB impairment.

### Neurotoxicity on immortalized human cell lines

#### Influence of MNP material, size, and charge on neurotoxicity and accumulation

Human neuroblastoma cell line SHSY-5Y, originating from the SK-N-SH cell subline, is a versatile in vitro model for neurotoxicity, ischemia, and neurological disorders, including Alzheimer’s disease and amyotrophic lateral sclerosis [353]. Studies showed that they respond differentially to PS MNPs based on size and surface charge. For instance, exposure to 25–50 nm PS and PS-NH₂ particles induced reduced neuronal differentiation, nuclear swelling, and morphological changes [281, 354]. MNPs localized predominantly to the cytoplasm, supporting their ability to cross cellular membranes [145].

#### Molecular and cellular toxicological impacts

Neurodegenerative processes were recapitulated in vitro, with increased aggregation of α-synuclein and enhanced secretion of amyloid β (Aβ), biomarkers strongly linked to Parkinson’s and Alzheimer’s disease [145, 149]. Additional studies using 70 and 150 nm PS MNPs confirmed that even at low doses, these particles could accelerate Aβ aggregation and elevate intracellular calcium levels and ROS generation, leading to cell damage [355].

#### Relevance to human disease

These findings suggest that MNP may not only contribute to cellular stress but also accelerate pathogenic processes associated with neurodegeneration. The ability of nano-sized plastic particles to enhance hallmark disease proteins like Aβ and α-synuclein warrants further exploration.

### Neurotoxicity on human pluripotent stem cells derived neural models

#### Influence of MNP material, size, and charge on neurotoxicity and accumulation

Human pluripotent stem cells (hPSCs) and their derivatives, including 2D neural cultures and 3D brain organoids, offer physiologically relevant platforms to model human neurodevelopment and disease. A recent study exposed cortical and sensory neurons to 20 nm, 100 nm, and 2 μm PS MNPs and polyester microfibers at environmentally relevant doses. Oxidative stress and neurodegeneration were observed in a size- and dose-dependent manner [356].

#### Exposure routes, bioaccumulation, and detection

In cerebral organoids, 50 nm particles penetrated deeper into tissue than 100 nm particles, suggesting that small size enables parenchymal infiltration and damage. After 21 days of exposure to 10 mg/mL particles, gene expression was significantly altered in pathways associated with neural viability and toxicity [306].

#### MNPs as vectors of toxicants

Importantly, biofilms formed by *Pseudomonas aeruginosa* and *Escherichia coli* on 2 μm PS MNPs exacerbated neurotoxicity [356], supporting the concept that MNPs may serve as biological vectors for pathogens or co-pollutants in the CNS [321].

#### Molecular and cellular toxicological impacts

Exposure to MNPs disrupted key neurodevelopmental processes, including altered proliferation, increased apoptosis, and disrupted tissue patterning in organoids and retinal models [357]. Long-term studies (4–30 days) on 3D forebrain models showed decreased expression of neural markers (Nestin, PAX6), DNA damage, and inhibition of cadherins, indicating potential impairment of neural circuit formation and neurodevelopment [358, 359].

#### Behavioral impairments and sexual dimorphism

While behavior cannot be directly assessed in vitro, altered gene expression in critical neurodevelopmental pathways can serve as a surrogate for long-term neurological dysfunction. The role of sex in these models remains unexplored, but future studies incorporating sex-specific hPSC lines or hormone treatments could shed light on this variable.

#### Relevance to human disease

These advanced human models offer unprecedented insight into how MNPs could impair human brain development and function. Findings suggest that environmentally relevant levels of MNPs may interfere with neurogenesis and brain architecture, raising important concerns for public health, particularly for vulnerable populations such as pregnant women and young children.

##  Lack of essential studies in the field

###  Protein corona

Although significant progress has been made in the field of MNPs over the past few decades, several critical studies are still lacking to fully understand the mechanisms by which biosystems respond to these particles, including the extent and nature of these responses. One of the most understudied yet pivotal aspects is the formation and composition of the protein corona on MNPs and how it influences their biodistribution, cellular uptake, and toxicity. This gap in knowledge hampers our ability to accurately predict the behavior and potential health risks associated with MNP exposure, particularly regarding their ability to cross biological barriers and accumulate in vulnerable tissues such as the nervous system.

Protein corona plays a vital role in determining the biological identity and behavior of MNPs within the human body. When NPs enter biological fluids, such as blood, proteins quickly adsorb onto their surface, forming a complex structure known as the protein corona [360, 361]. This corona effectively cloaks the original nanoparticle surface, influencing how the particles interact with cellular membranes, immune components, and biological barriers [362]. Of particular importance is the corona’s role in biodistribution. Once formed, it can modify the physicochemical properties of MNPs, affecting their stability, circulation time, and biodistribution within tissues. For MNPs to exert neurotoxic effects, they must pass through the BBB, a highly selective interface that protects the CNS from potentially harmful substances [363]. The composition of the protein corona can either facilitate or hinder the ability of NPs to cross this barrier, depending on the specific proteins adsorbed and the resulting surface characteristics. Understanding these interactions is essential for accurately predicting the neurotoxic potential of MNPs and their capacity to accumulate within neural tissues.

Despite the critical importance of the protein corona in mediating the biological effects of NPs, there remains a significant paucity of research in this area. To date, a few studies have systematically investigated the composition and dynamics of the protein corona formed on MNPs in human biological fluids, particularly within neural environments. This knowledge gap is problematic because the corona can drastically alter the particles’ toxicity, biodistribution, and ability to bypass biological barriers such as the BBB. Without detailed insights into the corona’s composition and behavior, it is challenging to predict which NPs may pose a higher risk of neurotoxicity or understand the mechanisms through which they might accumulate in neural tissues. Furthermore, the absence of standardized methodologies for studying the protein corona hampers cross-study comparisons and the development of comprehensive risk assessments.

The lack of empirical data on the protein corona’s formation and properties in human systems introduces significant limitations for risk evaluation and regulatory policymaking. Without this information, models of NPs’ behavior in the body remain incomplete, leading to potential underestimations of neurotoxic risks. Additionally, individual variations in proteomic profiles, health status, and environmental exposures further complicate the predictive capacity of current research, making it difficult to develop personalized risk assessments or effective mitigation strategies [364, 365]. In the broader context, neglecting the role of the protein corona in the biodistribution and neurotoxicity of MNPs impedes our understanding of how these particles interact with human tissues and how they can be controlled or prevented from causing harm. Advancing our knowledge in this area is crucial for creating accurate risk models, designing safer NPs, and ultimately protecting human health from the insidious risks posed by environmental MNPs.

### Error in toxicity assessments of MNPs

A significant challenge in the assessment of MNPs’ toxicity lies in the way their environmental concentrations are compared and interpreted. Currently, many toxicity studies base their data on pristine MNPs with well-characterized surface properties, often measured before these particles enter or interact with environmental conditions. However, this approach overlooks the dynamic nature of MNPs once they are released into the environment, where they are subject to various physical, chemical, and biological processes that alter their surface properties. Factors such as sunlight exposure, water chemistry, microbial activity, and interactions with other pollutants can lead to the formation of biofilms, surface oxidation, adsorption of environmental contaminants, or changes in surface charge. These modifications significantly influence the particles’ reactivity, stability, and toxicity profile.

The core issue stems from the fact that the toxicity data available are often derived from laboratory conditions that do not replicate the complex and variable environment in which MNPs are found. When researchers or policymakers compare environmental concentrations to toxicity thresholds established using pristine particles, they risk underestimating or misrepresenting the true risk. For example, surface modifications such as biofilm formation can enhance the uptake of MNPs by organisms or facilitate the crossing of biological barriers like the BBB, thus altering toxicity levels. Conversely, some environmental interactions may attenuate toxicity by reducing particle bioavailability or inducing surface passivation. Therefore, toxicity profiles based solely on unaltered MNPs may not accurately reflect real-world exposure risks.

This discrepancy points to the urgent need for studies that characterize MNPs directly extracted from environmental samples, accounting for their altered surface properties and contaminant loads. Only by understanding how environmental processes modify MNPs can we develop more accurate risk assessments and regulatory frameworks. This requires a shift towards environmentally relevant testing conditions and the integration of surface chemistry analysis in toxicity testing. Without these considerations, current assessments may significantly underestimate the potential health and ecological risks associated with MNPs, ultimately impeding effective policy development and mitigation strategies.

##  Conclusions and future considerations

MNP pollution has infiltrated the environment, raising concerns over its potential adverse effects on human health. Despite emerging studies on the possible harmful effects in terrestrial mammalian organisms caused by MNPs, there is a general inadequacy of data regarding their uptake and neurotoxicity. Escalating evidence indicates that MNPs can be taken up *via* different exposure routes by various organisms, including fish and rodents, resulting in elevated oxidative stress, inhibited AChE activity, altered neurotransmitter levels, and behavioral deficiencies in several species. However, whether these effects are related to human neurodevelopmental and neurodegenerative disorders, as shown for gold and metal nanoparticles, for instance, remains to be elucidated [366]. Here, we critically examined the current understanding of MNPs on environmental risks, pathways of exposure, neurotoxic effects, and the underlying mechanisms by comprehensively summarizing the existing research. This review offers valuable insights into MNP neurotoxicity and also emphasizes knowledge gaps and recommendations for future research (Table 2).

**Table 2:** Mechanisms of action with current evidence and knowledge gaps of MNPs exposure in higher vertebrates

Mechanisms	Evidence	Knowledge gaps
Disruption of Molecular Physiology	• Inhibited AChE [286, 290, 292, 304, 312, 329] • Mitochondrial Dysfunction [279, 303, 309, 330] • Disruption of Tight Junctions [279, 331] • Downregulated BDNF Gene Expression [280] • Oxidative Stress [279, 281, 284, 286, 287, 304, 308, 326, 327, 329] • Interaction with Tubb3 [367] • Activation of ASC-NLRP3-GSDMD Signaling Pathway [330] • Decreased ATP Levels [303, 316] • DNA Damage [284, 304] • Activation of TLR2/MMP9 Pathway [297] • Increase CREB/BDNF Pathway [287] • Reduction of Acetylcholine Levels [287] • Increased Lipid Peroxidation [287] • Activated NF-κB pathways [98] • Activated P53-mediated ferritinophagy [327]	• How exactly do MNPs or associated chemicals bind to or alter enzymes like AChE and disrupt mitochondrial function? • How do MNPs promote the generation of ROS? • Can MNPs exposure lead to heritable changes in gene expression or epigenetic modifications? • How does long-term MNPs accumulation affect the expression of key genes like BDNF, AChE, or mitochondrial genes? • Are there lasting impacts on DNA integrity, considering the reported DNA damage? • Are there threshold levels of exposure below which these pathways are not affected? • To what extent do the chemicals leached from MNPs (like bisphenol A, phthalates) contribute to the disruption of molecular pathways compared to the virgin particles themselves? • What are the upstream signals by which MNPs activate the NF-κB and the PI3K/AKT signaling and how do these pathways interact with oxidative stress and mitochondrial dysfunction observed in MNP exposure—are they parallel, compensatory, or synergistic?
Neural Damage	• Affected Neuronal Cyto-architecture [285, 296] • Reduced Nissle Bodies Counting in the Brain [296] • p53/BAX/BCL-2-Dependent Neuronal Apoptosis [297] • BBB Disruption [288, 297] • Ac-Tau-mediated Neurotoxicity [297] • Hippocampus Degeneration [290, 325, 326] • Impaired Hippocampal Neurogenesis [285, 299] • Impaired Hippocampal Neuroplasticity [300] • Altered Axonal Guidance [282] • Synaptic Disfunction [282, 284] • Altered Neurotrophin Signaling [282] • Loss of Purkinje Cells [330] • Microglia-Mediated Neuronal Damage [302] • Ferroptosis in the hippocampus [327]	• How do MNPs interfere with cytoskeletal elements (e.g., microtubules, actin filaments) that maintain neuronal structure? • How does prolonged MNPs exposure influence the balance between pro-apoptotic and anti-apoptotic signaling pathways? • Can MNPs cross the BBB directly, or are their neurotoxic effects mediated by secondary messengers such as cytokines? • How do MNPs interfere with synaptic vesicle trafficking, neurotransmitter release, or receptor function at synapses? • How do MNPs activate microglia, leading to neuroinflammatory responses, and what inflammatory mediators are involved? • What are the long-term impacts of MNPs exposure on brain health, and can early biomarkers be identified? • What molecular triggers link MNP exposure to ferroptosis in neurons? • Is ferroptosis a primary mechanism of neuronal loss in MNP-exposed brains, or does it occur secondary to other forms of stress (e.g., inflammation and mitochondrial dysfunction)?
Inflammation	• Increased Inflammatory Response [280, 287, 301–303, 306, 307, 310, 315, 319, 320]	• What are the specific molecular pathways activated by MNPs that lead to the initiation of inflammation? Are these pathways dependent on particle size, shape, or composition? • How do MNPs activate microglia in the brain, and what inflammatory mediators (e.g., cytokines, chemokines) are produced as a result? • What is the role of astrocytes in responding to MNPs accumulation, and how do they contribute to the inflammatory response? • Does MNPs-induced inflammation persist chronically, even after exposure ceases? If so, what are the mechanisms of sustained inflammation? • What are the key cytokines and chemokines released in response to MNPs exposure, both in peripheral tissues and the brain? Are there specific profiles for acute vs. chronic exposure? • How do plastic additives, such as bisphenols (BPA) or phthalates, influence the inflammatory response? Do these chemicals contribute to a more pronounced immune response than the plastic particles themselves?
Cognitive and Behavioral Deficits	• Memory Impairment [281, 286–289, 294, 320]	• To what extent is neuroinflammation, mediated by activated microglia and astrocytes, responsible for cognitive dysfunction and mood disorders (anxiety, depression) observed in MNPs-exposed animals?
	• Cognitive Dysfunction [281, 284, 286, 288, 289, 292, 304, 306, 311, 317, 327]	• Are specific inflammatory signaling pathways (e.g., NF-κB, NLRP3 inflammasome) involved in the onset of behavioral deficits?
	• Anxiety [279, 283, 286]	• Can antioxidant or anti-inflammatory treatments mitigate the behavioral effects of MNPs exposure, and what does this suggest about the causality of inflammation?
	• Impaired Novelty Preferences [324]	• Which brain regions (e.g., hippocampus, prefrontal cortex, cerebellum, amygdala) are most affected by MNPs accumulation, and how does this relate to specific behavioral outcomes like memory impairment, anxiety, and motor dysfunction?
	• Depression [279, 282]	• Does MNPs accumulation follow a region-specific pattern that mirrors behavioral deficits, such as hippocampal damage correlating with memory impairment?
	• Social Deficits [279, 324]	• Are the cognitive and emotional deficits caused by MNPs reversible upon cessation of exposure, or do they persist long-term?
	• Motor and Coordination Dysfunction [281]	• Are younger (developmental) or older (aging) individuals more vulnerable to behavioral deficits caused by MNPs exposure? How does age influence the severity of cognitive or motor dysfunction?
		• How do different sizes of MNPs (e.g., NPs vs. larger MPs) differentially affect behavior? Are smaller particles more likely to cross biological barriers (e.g., BBB) and cause greater neurobehavioral disruption?

**Fig. 7:**
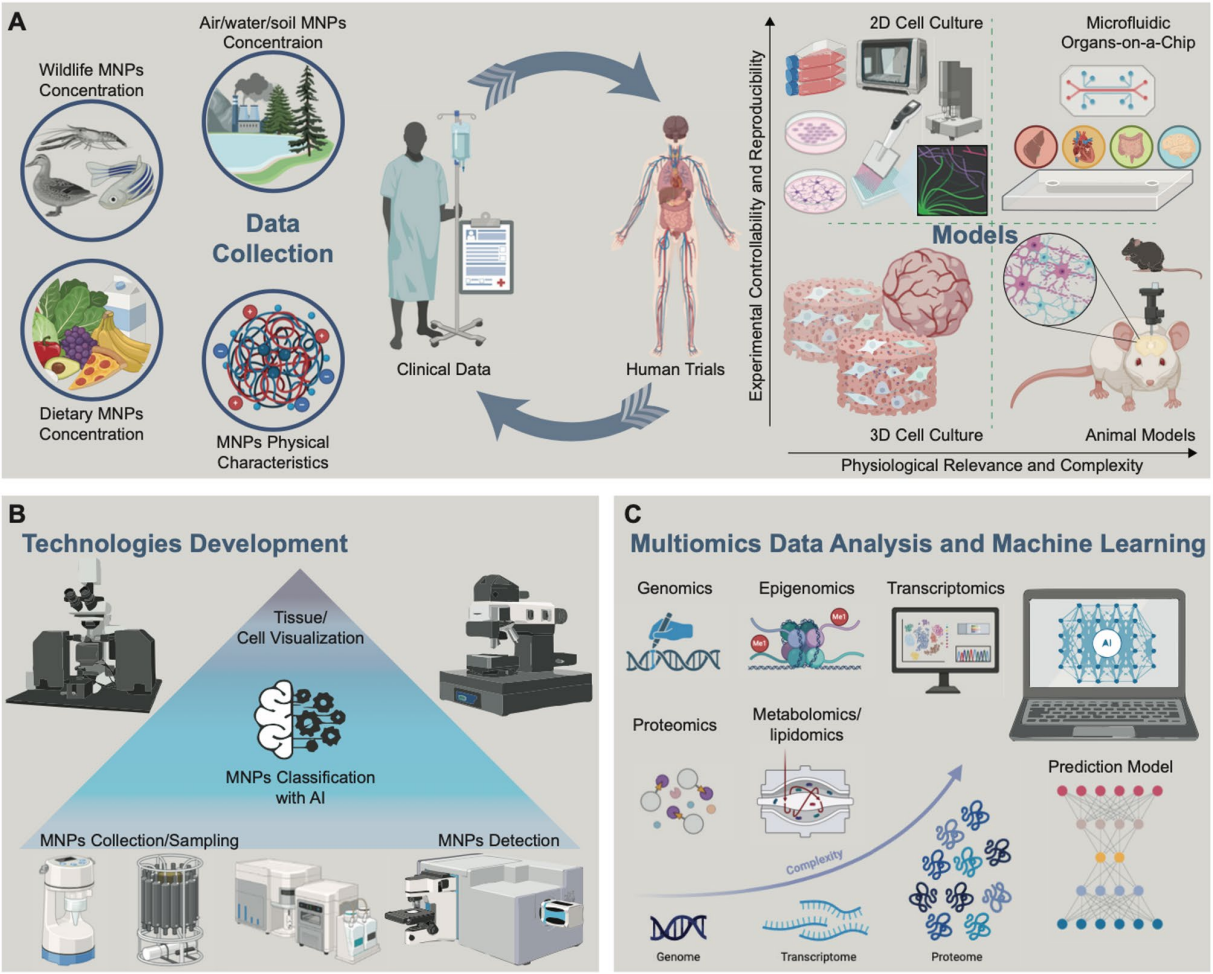
Future directions of hazard assessment of micro-and nanoplastics (MNPs). (A) Monitoring of human MNPs exposure levels requires real-time evaluation and data collection of MNPs concentration and characteristics from various environmental and clinical samples. To elucidate MNPs-induced neurotoxicity mechanisms, model systems development should include a wide spectrum of in vivo, in vitro, and/or human research study designs. (B) Technologies development focuses on sampling, collection, detection, and visualization methods capturing nano-sized particles. (C) Genomics, epigenomics, transcriptomics, proteomics, and metabolomics are complementary to each other, providing a comprehensive framework for research on MNPs’ neurotoxicity. The acquired data from different multiomics technologies, along with MNPs classification, should be converted to prediction models to understand the environmental and human health risks of MNPs exposure. Abbreviation: MNPs, micro-and nanoplastics; AI, artificial intelligence. *Created in BioRender. Vojnits*,* K. (2025)* https://BioRender.com/a57n112

Several actions are required, and inherent challenges need to be overcome to thoroughly investigate the neurotoxic hazard and risk of exposure to MNPs as listed below (Fig. 7):

1. The environment contains several types of MNPs, such as PE, PP, PVC, and PET, among others. These particles have complex sizes and shapes, including fiber, flake, sphere, fragment, and irregular shapes, while current studies have predominantly focused on sphere PS MNPs, neglecting the other polymers or shapes. Moreover, we determined that 83% of the neurotoxicity assays set concentrations much higher than the actual environmental levels, which is of little significance for toxicity assessment. Therefore, real evaluation data is needed, especially considering particle characteristics. These gaps warrant further investigation into the potential health impacts of these overlooked forms of MNPs, but for these, exposure levels, particularly for humans, require better monitoring. For this, besides improved sampling and detection methods of MNPs in the environment, novel and more precise analytical approaches (e.g., flow cytometry, light sheet microscopy, Fourier-transform infrared spectroscopy, Raman spectroscopy combined with artificial intelligence) need to be developed to quantify and identify all sizes and shapes of MNPs in various environmental and biological samples.
2. In the environment, MNPs frequently adsorb biofilms formed by pathogenic microorganisms and diverse pollutants encompassing organic matter and metal pollutants. If MNPs act as a vector carrying these into the brain, they will probably exert a pronounced toxicity due to synergistic effects. To this end, to advance hazard characterization of heterogeneous mixtures of particles, synergy exposure studies with surface contaminants should be considered. Moreover, to improve reliability, consistency, and comparability across studies, densities, particle numbers, and surface characteristics of applied MNPs should be reported.
3. Inhalation of plastic particles is one of the main exposure ways in the human body; however, despite an increase in toxicological studies on the neurotoxicity of MNPs in recent years, there is a scarcity of research examining the toxicological consequences of inhaling airborne MNPs on the brain. Further experiments are needed to unravel the effects and mechanisms of inhalable MNPs and their contribution to neurotoxicity.
4. Upon entering the body, MNPs could be transferred to diverse tissues, including the brain. Understanding their distributions, accumulation, and excretion after various exposure routes, i.e., inhalation, ingestion, and dermal contact, is the utmost priority in exploring their potentially harmful consequences. The absorption-distribution-metabolism-excretion process of MNPs and their precise migration and transformation pathways through which they can reach individual tissues remain unknown. The brain is a potential toxic target organ of MNPs, whereas explanations for MNPs entering the brain are mostly based on nanoparticle studies. Thus, more studies are warranted to validate the mechanisms by which internal plastics are transferred to the brain.
5. Environmental and biological transport of MNPs can significantly alter their physicochemical properties. The interaction of MNPs with substances in environmental and biological media may result in the formation of a biomolecular corona, a layer of biological molecules, on their surface. Given the hydrophobic, inert, and persistent features of MNPs, corona formation is a prominent process that is highly related to the migration, uptake, distribution, metabolism, clearance, and toxicity of MNPs. Full interpretation of a corona structure is highly challenging and requires a rational combination of different analytical methods. All considered, corona formation in MNPs safety assessment is a new research field that requires the exploration of interdisciplinary approaches in toxicology, materials science, and analytical chemistry. A full understanding of the impacts of the biomolecular corona on neurotoxicity may facilitate the establishment of a predictive toxicology paradigm for risk assessments of MNPs.
6. Studies on invertebrates, vertebrates, and cell-based in vitro models have demonstrated that the neurotoxic effects induced by MNPs traverse numerous regulatory pathways, including oxidative stress, inhibition of AChE, and immunoinflammatory response, among others. Nevertheless, the exact mechanism by which MNPs elicit neurotoxicity remains unknown. Subsequent investigations are suggested to incorporate a mechanism-based framework, such as the adverse outcome pathway, to classify and link these detrimental effects. Moreover, advanced 3D in vitro models, i.e., organoids and organs-on-a-chip models, coupled with multi-omics (metagenomics, transcriptomics, proteomics, metabolomics) and predictive modeling, e.g., with machine learning or AI, will deepen our understanding of MNPs-induced neurotoxicity mechanisms.
7. While substantial progress has been made in understanding the dynamics and impacts of MNPs, critical gaps remain that hinder accurate risk assessment and effective regulation. The formation and characterization of the protein corona, a key factor influencing MNP biodistribution, cellular interaction, and ability to cross biological barriers such as the blood-brain barrier, remains largely understudied. Without detailed knowledge of the corona’s composition and behavior in human systems, our ability to predict neurotoxicity and the potential for neural tissue accumulation is severely limited. Additionally, current toxicity assessments often rely on data obtained from pristine MNPs, neglecting the profound surface modifications these particles undergo in environmental settings. Such oversight can lead to underestimations of toxicity and misinformed policy decisions. Addressing these deficiencies through standardized methodologies, environmentally relevant testing, and comprehensive surface chemistry analysis is imperative. Doing so will improve our understanding of MNP behavior in realistic conditions, enabling more accurate risk models and the development of safer NPs, ultimately safeguarding human health against the insidious threats posed by environmental MNPs.
8. Importantly, the majority of MNP research lacks adequate quality control, reproducibility, and statistically robust sample sizes, thereby limiting the ability to reliably assess the real adverse effects of MNPs. As previously stated, more stringent standards, transparency, and collaboration among researchers, politicians, and industry stakeholders are required [368].
9. Many questions remain to be answered in the quest to understand the impact of MNPs on the human body, especially on the human brain. Most research has been conducted on cell lines, which overlook physiological processes, such as the body’s immune reaction to the entry of foreign substances. Detection methods or instruments to accurately measure the concentration of MNPs in the environment and within the body are yet to be defined, as the techniques are predominantly experimental. To understand the association, if there is any, of MNPs with neurodegenerative diseases, more clinical data regarding the MNPs concentration in patients is required. For this, potential confounders, such as age, sex, residence, nutritional status, comorbidity, generalized larger population, and simultaneous multiple outcome measurements, should also be taken into account in order to guarantee accurate observational studies. While observational and biomarker-based studies are required to obtain deep insights, studies on animals and cells can aid in understanding adverse effects and the mechanism of action.
10. NPs’ neurotoxicity has the potential to significantly influence human behavior by disrupting normal neural functions. As these tiny particles penetrate the BBB and accumulate in neural tissues, they may induce oxidative stress, inflammation, and cellular damage within the brain. Such neurotoxic effects can alter neurotransmitter levels, impair synaptic connectivity, and disrupt neural signaling pathways, potentially leading to cognitive deficits, mood disturbances, and behavioral changes. These alterations could manifest as increased anxiety, depression, impaired memory, or diminished decision-making abilities, ultimately affecting daily functioning and quality of life. Given the pervasive environmental presence of NPs, understanding their neurotoxic impact is crucial, as it may reveal subtle but far-reaching effects on mental health and behavior, especially with chronic exposure. Addressing this emerging issue is essential for developing preventative strategies and informing public health policies aimed at mitigating the neurobehavioral risks associated with NPs.In conclusion, only a transdisciplinary approach will enable researchers to better understand the environmental and human health risks of exposure to MNPs (Fig. 7).

## Supplementary Information

Below is the link to the electronic supplementary material.

Supplementary Material 1

Supplementary Material 2

## Data Availability

All data generated or analyzed during this study are included in this published article and its supplementary information files.
