# Supplementary material for: A systematic review of the potential neurotoxicity of micro-and nanoplastics: the known and unknown
Source: Part Fibre Toxicol. 2025 Nov 6;22:29. doi: 10.1186/s12989-025-00647-4 (PMC12590691; doi:10.1186/s12989-025-00647-4)
Supplement: Supplementary file 2 — Supplementary Material 2 [file 12989_2025_647_MOESM2_ESM.docx]

| MNPs type | MNPs Size | Animals | Exposure Route | Duration | Concentration  Per Dose | Neurotoxic Effects | Ref. |
| --- | --- | --- | --- | --- | --- | --- | --- |
| PS beads | 108 nm | C57BL/6 J mice,  male/female, 6 months, n= 6 per group | Oral admin | 30 days | 50 mg/kg | Neuroinflammation via activation of lipophagy-related lipolysis | Qian et al, 2025^1^ |
| PS beads | 0.05–0.1 µm | Sprague Dawley rats, female, pregnant, and offspring, n = 15 female and 15 male pups | Oral gavage | 4 days | 20 mg/kg/day | No direct changes in brain neurotransmitters or related metabolites;  Plasma metabolomic shifts in amino acid and lipid pathways | Mortensen et al, 2025^2^ |
| PS beads | NR | Sprague Dawley rats, female, pregnant, and offspring | Oral gavage | 43 days | 2.5 mg/kg | Spatial transcriptomics revealed fewer neurons, more astrocytes, and more excitatory neurons 1 in the offspring hippocampal region; decrease in C3-type astrocytes and an increase in C2-type astrocytes | Tian et al, 2025^3^ |
| PET beads | 83 nm | Swiss albino mice, male, mature, n = 10 per group | Oral gavage | 30 days | 200 mg/kg bw | Increase of IL-1ß and glial fibrillary acidic proteins in brain tissues;  Upregulated malondialdehyde, cyclooxygenase-2, and IL-1ß;  Downregulated glutathione and AChE | Kamel et al, 2025^4^ |
| PS beads | 5 µm  0.5 µm | C57BL/6 J mice,  male, 21 postnatal days, n= 10 per group | Oral admin | 4 weeks | 0.5 mg | Significant cognitive impairment;  Neuronal loss and neurogenesis inhibition;  Dendritic spine damage in the hippocampus;  Activation of the PI3K/AKT pathway | Wang et al, 2025^5^ |
| Beads (polymer type not specified) | 50 nm  100 nm | C57BL/6 mice,  male, 8 weeks, n = 3 per group | Oral admin | 7 days | 2.5 mg/mL | Accumulation in brain;  DNA fragmentation in the hippocampus and cortex; Elevated levels of inflam- matory markers and neurotoxic metabolites, such as kynurenine (KYN) and 3-hydroxykynurenine (3-HK) | Park et al, 2025^6^ |
| LDPE irregularly granular  Oxidized LDPE irregularly granular | 2.67 to 12.61 µm | C57BL/6 mice,  male, 6 weeks, n = 12 per group | Oral admin | 28 days | 5 mg/mL | Neurocognitive defects;  Alteration of cholinergic system;  AChE reduction | Wang et al, 2024^7^ |
| PS beads | 5 µm | C57BL/6 J mice,  male, 6 weeks, n= 8 per group | Intratracheal instillation | 60 days | 0.6 mg/kg  3 mg/kg  15 mg/kg | No accumulation in brain;  M1 polarization of microglia; Impaired cognitive function; Altered pulmonary flora with increased lipopolysaccharide production | Kang et al, 2024^8^ |
| PS beads | 50 nm | Sprague Dawley rats, female, pregnant, n = 5 per group | Oral gavage | 45 days | 2.5 mg/kg | Ferroptosis in the hippocampus of the offspring;  Declined cognitive, learning, and memory abilities;  ROS production;  Activated P53-mediated ferritinophagy | Chen et al, 2024^9^ |
| PS beads  PS-COOH beads  PS-NH_2_ beads | 80 nm | C57BL/6J mice,  male, 8 weeks,  n = 6 per group,  n = 18 per group | Intranasal admin  Gastric feeding  Intratracheal instillation | 7 days  28 days | 50 mg/kg bw  2.5, 5, 10 mg/kg bw | Brain accumulation;  Reduced exploratory and spatial learning ability;  Activated NF-κB pathways | Sun et al, 2024^10^ |
| Mainly PS fragments from disposable cups | 6 µm to 154 µm | C57BL/6J mice, female, n = 20 per group | Oral gavage | 18 days | 200 µL MP solution | Accumulation in the fetus, placenta, kidney, spleen, lung, heart, brain;  Reduced fetal development and growth;  Upregulated oxidative stress pathways;  Microbiome disturbances | Chen et al, 2024^11^ |
| PS beads | 50 nm | *Nrf2^fl/fl^*-*Vil^Cre+^* C57BL/6J mice,  male, 8 weeks,  n = 12 per group,  n = 45 per group | Oral ingestion | 28 days | 2.5 mg/kg  250 mg/kg | Gut microbiota dysbiosis;  Increased interleukin 17C;  Neuroinflammation | Lian et al, 2024 |
| PS beads | 30 – 50 nm | C57BL/6J mice,  male, 7 weeks,  n = 6 per group | Oral ingestion | 6 weeks  7 weeks | 10 mg/kg  20 mg/kg | Accumulation in brain;  Memory impairment;  Cognitive dysfunction | Paing et al,  2024^12^ |
| PS beads  PS-COOH beads  PS-NH_2_ beads | 100 nm | BALB/c mice,  male, 4 weeks,  n = 32 in total | Oral gavage | 28 days | 1 mg (1.8 × 10^12^ particles) | Accumulation in brain;  Anxiety, depression, social deficits;  Disruption of tight junctions;  Mitochondrial dysfunction | Ma et al, 2024^13^ |
| PS beads | 480 ± 30.3 nm | C57BL/6J mice,  male, 8 weeks | Oral gavage | 7 days | 2 mg/kg  10 mg/kg | Anxiety;  Pro-inflammatory response through the HRAS-induced PERK- NF-κB pathway | Li et al, 2024^14^ |
| PS beads  PE beads | 5 µm  1-4 µm | C57BL/6J mice, male and female, 8-12 weeks, n = 8 per group | Oral gavage | Twice a week for 4 weeks | 2 or 4 mg/week | PS beads detected in brain, liver, and kidney;  Concentration- and particle type-dependent metabolic changes in colon, liver, and brain | Garcia et al, 2024^15^ |
| PS carboxylate modified beads | 500 nm | Swiss albino mice,  male,  n = 5 per group, repeated 3 times | Oral gavage | 28 days | 0.1 ppm  1 ppm  10 ppm | Accumulation in brain;  Reduced Nissl bodies in the brain;  Affected neuronal cyto-architecture;  Downregulated BDNF gene expression | Suman et al, 2024^16^ |
| PS beads | 100 nm | C57BL/6J mice,  pregnant female, 14 days male offspring, n = 3 per group | Oral gavage | 32 days | 50 μg/mL | Increased microglia and brain TNF-α level;  Dysregulated dopamine and serotonin metabolism;  Depletion of anti-inflammatory, and enriched pro-inflammatory bacteria | Li et al, 2024^17^ |
| PLA polymer beads  PLA oligomer beads | 100 nm | C57BL/6J mice,  male, 6 weeks,  n = 8 or 18 per group | Oral gavage | 28 days | 2.5 mg/kg  25 mg/kg | Bioaccumulation in blood, brain, liver, spleen, lungs, kidneys, and epididymis;  Upregulation of MICU3 in midbrains;  Neuronal mitochondrial calcium overload | Liang et al, 2024^18^ |
| PS beads | 500 nm | C57BL/6J mice,  male, 7-8 weeks,  n = 10 per group | Oral gavage | 30 days | 5 mg/kg | Perturbed gut microbiota homeostasis;  Brain lesions and inflammation;  Declined leaning and memory abilities | Sun et al, 2024^19^ |
| PS beads | 5 µm  100 nm | ICR mice,  male/female, 5 weeks,  n = 8 per group | Oral gavage | 90 days | 0.3 mg/kg  0.03 mg/kg | Aggravated toxicity of silver nanoparticles;  Altered energy, choline metabolisms;  Fatty acid peroxidation and inflammatory responses;  Microbial abundances changes | Zhang et al, 2024^20^ |
| PS beads | 200 nm  800 nm | C57BL/6J mice,  female,  n = 9 per group | Oral gavage | 4 weeks | 10^9^ particles | Altered microbiota profile, *Firmicutes*/*Bacteroidetes*;  Induced metabolic perturbations in gut and brain;  MP-microbiota-metabolite relationship | Lee et al, 2024^21^ |
| APS (amino-modified PS) | 100 nm | C57BL/6J mice,  male, 6-8 weeks,  n = 6 or 8 per group | Oral gavage | 15 weeks | 40 mg/kg | Accumulation in brain;  BBB impairment through TLR2/MMP9 signaling;  Neuronal apoptosis via the p53/Bax/Bcl-2 axis;  Neurotoxicity by increased neuronal Ac-Tau expression;  Camellia Pollen improved neurotoxicity via GAPDH/  CBP/Ac-Tau axis | Bai et al, 2024^22^ |
| PS beads | 2 µm | C57BL/6J mice,  female, 8 weeks,  n = 3 per group | Intranasal injection | 28 days | 0.68 mg/kg bw (1.2 × 10^6^ particles) | Accumulation in brain | Vojnits et al,  2024^23^ |
| PS beads | 2 µm | C57BL/6J mice,  offspring,  female and male,  n = 19 per group | Drinking water | From embryonic day 9.5 to postnatal day 168 | 1 mg/L | Accumulation in the digestive and excretory organs but not in the brain;  Impaired social novelty preferences | So et al,  2023^24^ |
| PS beads | 50 nm | Swiss albino mice,  male, 10 weeks,  n = 18 per group | Oral gavage | 8 weeks | 0.2 mg/mL  1 mg/mL | Anxiety, decreased cognitive function and memory;  Oxidative stress;  Decreased expression of neurotransmitter-related genes;  Alterations in antioxidant enzymes, AChE activities | Sharma et al,  2023^25^ |
| PS beads | 0.1 µm  2 µm | C57BL/6J mice,  female, 4 months, 21 months,  n = 10 per group | Drinking water | 3 weeks | 0.0025 mg/mL  0.025 mg/mL  0.125 mg/mL | Alterations in open-field and light-dark preference tests;  Immune marker changes in brain tissues;  Neurobehavioral and immune alterations varied between young and old mice | Gaspar et al, 2023^26^ |
| PS beads | 50 nm | C57BL/6J mice,  male, adult,  n = 10 per group | Oral gavage | 28 days | 250 mg/kg | Motor and coordination impairments prevented by melatonin by mitigating dopaminergic neurons’ mitophagy via the regulation of the AMPK/ULK1 pathway | Huang et al,  2023^27^ |
| PP beads | 7-20 µm | ICR mice,  male, 3 weeks,  n = 15 per group | Oral gavage | 28 days | 5 mg/kg (1.58 × 10^6^ particles) | Neurocognitive and memory deficit;  Damage to the CA3 region of the hippocampus;  Increased oxidative stress; Decreased AChE activity | Yang G et al,  2023^28^ |
| PS beads  PS-NH_3_^+^ beads  PS-COO^-^ beads | 50 nm  50 nm, 2000 nm  30 nm, 2000 nm | C57BL/6J mice,  male, 5 weeks | Oral admin | 10 days | 50 mg/kg  200 mg/kg | Only PS-NH3^+^ impaired hippocampal neurogenesis via reduced cell proliferation | Yang S et al,  2023^29^ |
| PS prepared in a ball mill | 17 nm | Wistar albino rats,  female and male, 6-8 weeks,  n = 5f/5m per group | Oral gavage | 4 weeks | 25 mg/kg  50 mg/kg | Accumulation in the hippocampi;  Neuronal degeneration in the hippocampi;  Significant differences in peroxidase (POD) and glutathione S-transferase (GST) values and fecal boli and grooming numbers in female rats | Bas et al,  2023^30^ |
| PS beads | 80 nm | C57BL/6J mice,  male, 6 weeks,  n = 9 per group | Oral gavage | 42 days | 60 µg | Accumulation in brain and gut;  Decreased hippocampal neuroplasticity;  Neurotoxicity;  Involvement of gut-brain axis through circadian rhythm-related pathways;  Melatonin or probiotics rescued neurological damage | Kang et al,  2023^31^ |
| PS beads  PS weathered beads | 100 µm | C57BL/6J mice,  male, 6 weeks,  n = 3 per group | Oral gavage | 1 week | 0.1 µg/g | Weathered beads increased activation of immune and neurodegeneration- related pathways;  More severe inflammatory responses | Kim et al,  2023^32^ |
| PS beads | 100 nm | CD-1 (ICR) mice,  male, 4-5 weeks, | Intranasal injection | 3 hours | 200 mg/L | Accumulation in brain;  Co-localization with Tubb3 neurons | Han et al, 2023^33^ |
| PS beads | 500 nm  5 µm  50 µm | Xinghua chicken,  30 days,  n = 24 per group | Oral gavage | 1, 7, 14, 21, 28, 35 days | 0.5 mg/mL | MNPs residue detected in small intestines, liver, skeletal muscles;  Inhibited energy and lipid metabolism;  Induced oxidative stress;  Decreased AChE activity in skeletal muscle;  Metabolomic profile change;  Reduced meat quality | Chen et al, 2023^34^ |
| PS beads | 25 nm | C57BL/6J mice,  male, 5 weeks,  n = 10 per group | Oral gavage | 6 months | 10 mg/kg bw  25 mg/kg bw  50 mg/kg bw | Depression-like responses in highest exp group;  Altered expression of 116 lncRNAs, 29 miRNAs, 987 mRNAs;  Effected axon guidance, dopaminergic synapses, and neurotrophin signaling pathways | Liu et al, 2023^35^ |
| PS beads | 1 µm  100 nm | C57BL/6J mice,  male, 6-8 weeks,  n = 5 per group | Oral gavage | 60 days | 0.5 mg | Anxiety;  Damaged intestinal mucosal barrier and increased permeability;  Intestinal microbiota dysbiosis;  Metabolism disorder | Chen et al, 2023^36^ |
| PS beads | 50 nm  500 nm  5 µm | C57BL/6N mice,  male, 3 weeks,  n = 5 per group | Intragastrical admin | 90 days | 50 mg/kg bw | Entering the blood system;  Different distribution in organs by particle size;  Increased inflammatory factors | Han et al, 2023^37^ |
| PS beads | 5 µm | Chickens,  female/male, 1 day,  n = 15f/15m per group | Drinking water | 42 days | 1 mg/L  10 mg/L  100 mg/L | Cerebral hemorrhage;  Generation of microthrombi;  Loss of Purkinje cells;  Disrupted mitochondrial function;  Activated ASC-NLRP3-GSDMD signaling pathway | Yin et al, 2022^38^ |
| PS beads | 500 nm  4 µm  10 µm | SPF Balb/c mice,  male, 6 weeks,  n = 12 per group | Drinking water | 180 days | 100 µg/L (1.437 × 10^9^ particles/L)  1000 µg/L (1.437 × 10^10^ particles/L)  100 µg/L (2.986 × 10^6^ particles/L)  1000 µg/L (2.986 × 10^7^ particles/L)  100 µg/L (1.737 × 10^5^ particles/L)  1000 µg/L (1.737 × 10^6^ particles/L) | Brain accumulation;  BBB disruption;  Cognitive and memory deficits | Jin et al, 2022^39^ |
| PS beads | 1-10 µm | SPF Kunming mice,  male, 6 weeks,  n = 15 per group | Food and drinking water | 30 days | 10 mg/L | Swelling and rupture of mitochondria in the cerebra;  Significant decrease in ATP and ATP levels | Zhang et al,  2022^40^ |
| PS beads | 5 µm | Chickens,  1 day,  n = 30 per group | Drinking water | 6 weeks | 1 mg/L  10 mg/L  100 mg/L | Liver metabolism disorders and increased glutamine and glutamate synthesis;  Reduced expression of tight junction proteins in BBB;  Reduced number of Purkinje cells | Yin et al, 2022^41^ |
| PS beads | 25 nm | C57BL/6 mice,  male, 5 weeks,  n = 10 per group | Oral gavage | 6 months | 10 mg/kg bw  25 mg/kg bw  50 mg/kg bw | Dose-dependent cognitive decline;  Increased ROS levels and DNA damage;  95 mRNAs altered associated with synaptic dysfunction | Chu et al,  2022^42^ |
| PS beads | 42 nm | C57BL/6J mice,  male, 8 weeks | Oral gavage | 7 days | 0.5 mg/kg bw  2.5 mg/kg bw  10 mg/kg bw  50 mg/kg bw | MNPs brain accumulation;  Induced microglia activation and neuron damage;  Induced oxidative stress;  Inflammatory response | Shan et al,  2022^43^ |
| PS beads | 50 nm | C57BL/6J mice,  male, adult | Oral gavage | 28 days | 0.25 mg/kg bw  2.5 mg/kg bw  25 mg/kg bw  250 mg/kg bw | Observed Parkinson’s disease-like neurodegeneration;  Energy metabolism disorder and mitochondrial dysfunction;  Inflammatory turbulence in astrocytes and microglia;  Decreases in ATP content | Liang et al,  2022^44^ |
| PS beads | 5.0–5.9 μm | Chinese Kun Ming mice,  male, 5 weeks,  n = 10 per group | Oral gavage | 4 weeks | 0.01 mg  0.1 mg  1 mg | Impaired learning and memory functions;  Acetylcholine level reduction;  Oxidative stress;  Inhibited CREB/BDNF pathway;  Vitamin E treatment restored the learning and memory abilities and rebounded the release of neuro- transmitters | Wang et al,  2022^45^ |
| PS beads | 5 µm | C57BL/6 mice,  male, 9 months,  n = 7 per group | Drinking water | 3 months | 1000 μg/L | MNPs in cortex and hippocampus;  Elevated lipid peroxidation and inflammation;  Cognitive impairment | Liu et al,  2022^46^ |
| PS beads  PS-COOH beads  PS-NH_2_ beads | 80 nm | Balb/c mice,  5-6 weeks,  n = 6 per group | Aerosol inhalation | 7 days | 1 mg | Brain and liver deposition;  Inhibition of AChE activities | Liu et al,  2022^47^ |
| PS beads | 200 nm  2 μm  10 μm | C57BL/6 mice,  8 weeks,  n = 6 per group | Oral admin | 24 hours  7 days | 2.5 μg/mL  10 μg/mL | Cortex, hippocampus, cerebellum deposition;  Brain-deposited MPs co-localized with microglia marker Iba1 | Kwon et al,  2022^48^ |
| PS beads  PS-COOH^-^ beads | 50 nm  500 nm | C57BL/6J mice,  female, pregnant | Oral admin | From embryonic day 8 to postnatal day 14 | 0.5–1000 μg/day | Brain accumulation;  Altered functioning of NSCs, neural cell compositions, and brain histology in progeny; | Jeong et al,  2022^49^ |
| PS beads  PS-ZnO beads | 23 nm | Swiss mice,  male, 4-6 weeks,  n = 12 per group | Intraperitoneal admin | 3 days | 14.6 ng/kg bw | Brain accumulation of MNPs;  Cognitive impairment;  Increased NO and TBA levels;  Suppressed AChE activity;  Erythrocyte DNA damages | Estrela et al,  2021^50^ |
| PS beads  PS-COOH beads  PS-NH_2_ beads | 100 nm | Balb/c mice,  male, 6 weeks,  n = 6 per group | Oral gavage | 28 days | 10 mg/mL (100 μL) | Accumulation in spleen, lung, kidney, intestine, testis, brain | Xu et al,  2021^51^ |
| PS beads | 5 μm  20 μm | ICR mice,  male, 5 weeks, n = 5 per group | Oral gavage | 1, 2, 4, 7, 14, 21, 28 days | 0.1 mg/day | Size-dependent accumulation in lover, kidney, and gut;  Disturbed energy and lipid metabolism pathways;  Oxidative stress;  Altered blood biomarkers of neurotoxicity | Deng et al, 2017^52^ |

1. Qian, B., Wang, C.Q., Su, Z., Jiang, R.J., Zhang, Z.Y., Che, L., and Song, J.L. (2025). FGF1 alleviates polystyrene nanoplastics-induced neuroinflammation through the suppression of lipophagy. Int J Biol Macromol *302*, 140531. 10.1016/j.ijbiomac.2025.140531.

2. Mortensen, N.P., Caffaro, M.M., Krovi, A., Kim, J., Watson, S.L., Snyder, R.W., Patel, P.R., Fennell, T.R., and Johnson, L.M. (2025). Oral Exposure to Nylon-11 and Polystyrene Nanoplastics During Early-Life in Rats. Nanomaterials (Basel) *15*. 10.3390/nano15060465.

3. Tian, L., Chen, J., Liu, X., Wei, Y., Zhao, Y., Shi, Y., Li, K., Liu, H., Lai, W., and Lin, B. (2025). Prenatal exposure on nanoplastics: A study of spatial transcriptomics in hippocampal offspring. Environ Pollut *366*, 125480. 10.1016/j.envpol.2024.125480.

4. Kamel, N.A., Bashir, D.W., El-Leithy, E.M.M., Tohamy, A.F., Rashad, M.M., Ali, G.E., and El-Saba, A.A.A. (2025). Polyethylene terephthalate nanoplastics-induced neurotoxicity in adult male Swiss albino mice with amelioration of betaine: a histopathological, neurochemical, and molecular investigation. Naunyn Schmiedebergs Arch Pharmacol. 10.1007/s00210-025-03867-9.

5. Wang, C., Lin, K., Zhang, Z., Pan, Y., Miao, Q., Han, X., Zhang, Z., Zhu, P., Yang, J., Peng, Y., et al. (2025). Adolescent exposure to micro/nanoplastics induces cognitive impairments in mice with neuronal morphological damage and multi-omic alterations. Environ Int *197*, 109323. 10.1016/j.envint.2025.109323.

6. Park, S.B., Jo, J.H., Kim, S.S., Jung, W.H., Bae, M.A., Koh, B., and Kim, K.Y. (2025). Microplastics Accumulation Induces Kynurenine-Derived Neurotoxicity in Cerebral Organoids and Mouse Brain. Biomol Ther (Seoul). 10.4062/biomolther.2024.185.

7. Wang, J., Yang, Y., Shi, Y., Wei, L., Gao, L., and Liu, M. (2024). Oxidized/unmodified-polyethylene microplastics neurotoxicity in mice: Perspective from microbiota-gut-brain axis. Environ Int *185*, 108523. 10.1016/j.envint.2024.108523.

8. Kang, H., Huang, D., Zhang, W., Wang, J., Liu, Z., Wang, Z., Jiang, G., and Gao, A. (2024). Pulmonary Flora-Derived Lipopolysaccharide Mediates Lung-Brain Axis through Activating Microglia Involved in Polystyrene Microplastic-Induced Cognitive Dysfunction. Adv Sci (Weinh) *11*, e2404966. 10.1002/advs.202404966.

9. Chen, J., Yan, L., Zhang, Y., Liu, X., Wei, Y., Zhao, Y., Li, K., Shi, Y., Liu, H., Lai, W., et al. (2024). Maternal exposure to nanopolystyrene induces neurotoxicity in offspring through P53-mediated ferritinophagy and ferroptosis in the rat hippocampus. J Nanobiotechnology *22*, 651. 10.1186/s12951-024-02911-9.

10. Sun, M., Zhang, M., Di, F., Bai, W., Sun, J., Zhang, M., Sun, J., Li, M., and Liang, X. (2024). Polystyrene nanoplastics induced learning and memory impairments in mice by damaging the glymphatic system. Ecotoxicol Environ Saf *284*, 116874. 10.1016/j.ecoenv.2024.116874.

11. Chen, Q., Peng, C., Xie, R., Xu, H., Su, Z., Yilihan, G., Wei, X., Yang, S., Shen, Y., Ye, C., and Jiang, C. (2024). Placental and fetal enrichment of microplastics from disposable paper cups: implications for metabolic and reproductive health during pregnancy. J Hazard Mater *478*, 135527. 10.1016/j.jhazmat.2024.135527.

12. Paing, Y.M.M., Eom, Y., Song, G.B., Kim, B., Choi, M.G., Hong, S., and Lee, S.H. (2024). Neurotoxic effects of polystyrene nanoplastics on memory and microglial activation: Insights from in vivo and in vitro studies. Sci Total Environ *924*, 171681. 10.1016/j.scitotenv.2024.171681.

13. Ma, Y., Xu, D., Wan, Z., Wei, Z., Chen, Z., Wang, Y., Han, X., and Chen, Y. (2024). Exposure to different surface-modified polystyrene nanoparticles caused anxiety, depression, and social deficit in mice via damaging mitochondria in neurons. Sci Total Environ *919*, 170739. 10.1016/j.scitotenv.2024.170739.

14. Li, G., Liu, X., Sun, X., Huang, L., Kuang, W., Ou, J., Zhang, J., Zhang, Z., Li, H., Tang, H., et al. (2024). Polystyrene microplastics induce anxiety via HRAS derived PERK-NF-kappaB pathway. Environ Int *185*, 108543. 10.1016/j.envint.2024.108543.

15. Garcia, M.M., Romero, A.S., Merkley, S.D., Meyer-Hagen, J.L., Forbes, C., Hayek, E.E., Sciezka, D.P., Templeton, R., Gonzalez-Estrella, J., Jin, Y., et al. (2024). In Vivo Tissue Distribution of Polystyrene or Mixed Polymer Microspheres and Metabolomic Analysis after Oral Exposure in Mice. Environ Health Perspect *132*, 47005. 10.1289/EHP13435.

16. Suman, A., Mahapatra, A., Gupta, P., Ray, S.S., and Singh, R.K. (2024). Polystyrene microplastics induced disturbances in neuronal arborization and dendritic spine density in mice prefrontal cortex. Chemosphere *351*, 141165. 10.1016/j.chemosphere.2024.141165.

17. Li, X., He, E., Chen, G., Cao, X., Zhao, L., Xu, X., Fu, Z., and Qiu, H. (2024). Intergenerational neurotoxicity of polystyrene nanoplastics in offspring mice is mediated by dysfunctional microbe-gut-brain axis. Environ Int *192*, 109026. 10.1016/j.envint.2024.109026.

18. Liang, B., Deng, Y., Zhong, Y., Chen, X., Huang, Y., Li, Z., Huang, X., Yang, X., Du, J., Ye, R., et al. (2024). Gastrointestinal Incomplete Degradation Exacerbates Neurotoxic Effects of PLA Microplastics via Oligomer Nanoplastics Formation. Adv Sci (Weinh) *11*, e2401009. 10.1002/advs.202401009.

19. Sun, H., Yang, B., Zhu, X., Li, Q., Song, E., and Song, Y. (2024). Oral exposure of polystyrene microplastics and doxycycline affects mice neurological function via gut microbiota disruption: The orchestrating role of fecal microbiota transplantation. J Hazard Mater *467*, 133714. 10.1016/j.jhazmat.2024.133714.

20. Zhang, L., Li, Q., Ding, S., Wei, Z., and Ma, Y. (2024). Biotoxicity of silver nanoparticles complicated by the co-existence of micro-/nano-plastics. Food Chem Toxicol *193*, 115020. 10.1016/j.fct.2024.115020.

21. Lee, S.H., Lin, W.Y., and Cheng, T.J. (2024). Microbiota-mediated metabolic perturbations in the gut and brain of mice after microplastic exposure. Chemosphere *350*, 141026. 10.1016/j.chemosphere.2023.141026.

22. Bai, H., Wu, Y., Li, H., Zhu, Y., Che, R., Wang, F., and Zhang, C. (2024). Cerebral neurotoxicity of amino-modified polystyrene nanoplastics in mice and the protective effects of functional food Camellia pollen. Sci Total Environ *912*, 169511. 10.1016/j.scitotenv.2023.169511.

23. Vojnits, K., de Leon, A., Rathore, H., Liao, S., Zhao, M., Gibon, J., and Pakpour, S. (2024). ROS-dependent degeneration of human neurons induced by environmentally relevant levels of micro- and nanoplastics of diverse shapes and forms. J Hazard Mater *469*, 134017. 10.1016/j.jhazmat.2024.134017.

24. So, Y.H., Shin, H.S., Lee, S.H., Moon, H.J., Jang, H.J., Lee, E.H., and Jung, E.M. (2023). Maternal exposure to polystyrene microplastics impairs social behavior in mouse offspring with a potential neurotoxicity. Neurotoxicology *99*, 206-216. 10.1016/j.neuro.2023.10.013.

25. Sharma, A., Kaur, M., Sharma, K., Bunkar, S.K., John, P., and Bhatnagar, P. (2023). Nano polystyrene induced changes in anxiety and learning behaviour are mediated through oxidative stress and gene disturbance in mouse brain regions. Neurotoxicology *99*, 139-151. 10.1016/j.neuro.2023.10.009.

26. Gaspar, L., Bartman, S., Coppotelli, G., and Ross, J.M. (2023). Acute Exposure to Microplastics Induced Changes in Behavior and Inflammation in Young and Old Mice. Int J Mol Sci *24*. 10.3390/ijms241512308.

27. Huang, Y., Liang, B., Li, Z., Zhong, Y., Wang, B., Zhang, B., Du, J., Ye, R., Xian, H., Min, W., et al. (2023). Polystyrene nanoplastic exposure induces excessive mitophagy by activating AMPK/ULK1 pathway in differentiated SH-SY5Y cells and dopaminergic neurons in vivo. Part Fibre Toxicol *20*, 44. 10.1186/s12989-023-00556-4.

28. Yang, G., Gong, C., Zheng, X., Hu, F., Liu, J., Wang, T., Chen, X., Li, M., Zhu, Z., Zhang, L., and Li, R. (2023). Early clues and molecular mechanism involved in neurodegenerative diseases induced in immature mice by combined exposure to polypropylene microplastics and DEHP. Environ Pollut *336*, 122406. 10.1016/j.envpol.2023.122406.

29. Yang, S., Lee, S., Lee, Y., Cho, J.H., Kim, S.H., Ha, E.S., Jung, Y.S., Chung, H.Y., Kim, M.S., Kim, H.S., et al. (2023). Cationic nanoplastic causes mitochondrial dysfunction in neural progenitor cells and impairs hippocampal neurogenesis. Free Radic Biol Med *208*, 194-210. 10.1016/j.freeradbiomed.2023.08.010.

30. Bas, O., Ilhan, H., Hanci, H., Celikkan, H., Ekinci, D., Degermenci, M., Karapinar, B.O., Warille, A.A., Cankaya, S., and Ozkasapoglu, S. (2023). To what extent are orally ingested nanoplastics toxic to the hippocampus in young adult rats? J Chem Neuroanat *132*, 102314. 10.1016/j.jchemneu.2023.102314.

31. Kang, H., Zhang, W., Jing, J., Huang, D., Zhang, L., Wang, J., Han, L., Liu, Z., Wang, Z., and Gao, A. (2023). The gut-brain axis involved in polystyrene nanoplastics-induced neurotoxicity via reprogramming the circadian rhythm-related pathways. J Hazard Mater *458*, 131949. 10.1016/j.jhazmat.2023.131949.

32. Kim, H.Y., Ashim, J., Park, S., Kim, W., Ji, S., Lee, S.W., Jung, Y.R., Jeong, S.W., Lee, S.G., Kim, H.C., et al. (2023). A preliminary study about the potential risks of the UV-weathered microplastic: The proteome-level changes in the brain in response to polystyrene derived weathered microplastics. Environ Res *233*, 116411. 10.1016/j.envres.2023.116411.

33. Han, S.W., Kim, T.Y., Bae, J.S., Choi, J., and Ryu, K.Y. (2023). Alleviation of neurotoxicity induced by polystyrene nanoplastics by increased exocytosis from neurons. Biochem Biophys Res Commun *668*, 19-26. 10.1016/j.bbrc.2023.05.070.

34. Chen, J., Chen, G., Peng, H., Qi, L., Zhang, D., Nie, Q., Zhang, X., and Luo, W. (2023). Microplastic exposure induces muscle growth but reduces meat quality and muscle physiological function in chickens. Sci Total Environ *882*, 163305. 10.1016/j.scitotenv.2023.163305.

35. Liu, Q., Hu, W., Zhang, Y., Ning, J., Pang, Y., Hu, H., Chen, M., Wu, M., Wang, M., Yang, P., et al. (2023). Comprehensive Analysis of lncRNA-mRNA Expression Profiles in Depression-like Responses of Mice Related to Polystyrene Nanoparticle Exposure. Toxics *11*. 10.3390/toxics11070600.

36. Chen, X., Xu, L., Chen, Q., Su, S., Zhuang, J., and Qiao, D. (2023). Polystyrene micro- and nanoparticles exposure induced anxiety-like behaviors, gut microbiota dysbiosis and metabolism disorder in adult mice. Ecotoxicol Environ Saf *259*, 115000. 10.1016/j.ecoenv.2023.115000.

37. Han, J., Yan, J., Li, K., Lin, B., Lai, W., Bian, L., Jia, R., Liu, X., and Xi, Z. (2023). Distribution of Micro-Nano PS, DEHP, and/or MEHP in Mice and Nerve Cell Models In Vitro after Exposure to Micro-Nano PS and DEHP. Toxics *11*. 10.3390/toxics11050441.

38. Yin, K., Lu, H., Zhang, Y., Hou, L., Meng, X., Li, J., Zhao, H., and Xing, M. (2022). Secondary brain injury after polystyrene microplastic-induced intracerebral hemorrhage is associated with inflammation and pyroptosis. Chem Biol Interact *367*, 110180. 10.1016/j.cbi.2022.110180.

39. Jin, H., Yang, C., Jiang, C., Li, L., Pan, M., Li, D., Han, X., and Ding, J. (2022). Evaluation of Neurotoxicity in BALB/c Mice following Chronic Exposure to Polystyrene Microplastics. Environ Health Perspect *130*, 107002. 10.1289/EHP10255.

40. Zhang, W., Sun, X., Qi, X., Liu, X., Zhang, Y., Qiao, S., and Lin, H. (2022). Di-(2-Ethylhexyl) Phthalate and Microplastics Induced Neuronal Apoptosis through the PI3K/AKT Pathway and Mitochondrial Dysfunction. J Agric Food Chem *70*, 10771-10781. 10.1021/acs.jafc.2c05474.

41. Yin, K., Wang, D., Zhao, H., Wang, Y., Zhang, Y., Liu, Y., Li, B., and Xing, M. (2022). Polystyrene microplastics up-regulates liver glutamine and glutamate synthesis and promotes autophagy-dependent ferroptosis and apoptosis in the cerebellum through the liver-brain axis. Environ Pollut *307*, 119449. 10.1016/j.envpol.2022.119449.

42. Chu, C., Zhang, Y., Liu, Q., Pang, Y., Niu, Y., and Zhang, R. (2022). Identification of ceRNA network to explain the mechanism of cognitive dysfunctions induced by PS NPs in mice. Ecotoxicol Environ Saf *241*, 113785. 10.1016/j.ecoenv.2022.113785.

43. Shan, S., Zhang, Y., Zhao, H., Zeng, T., and Zhao, X. (2022). Polystyrene nanoplastics penetrate across the blood-brain barrier and induce activation of microglia in the brain of mice. Chemosphere *298*, 134261. 10.1016/j.chemosphere.2022.134261.

44. Liang, B., Huang, Y., Zhong, Y., Li, Z., Ye, R., Wang, B., Zhang, B., Meng, H., Lin, X., Du, J., et al. (2022). Brain single-nucleus transcriptomics highlights that polystyrene nanoplastics potentially induce Parkinson's disease-like neurodegeneration by causing energy metabolism disorders in mice. J Hazard Mater *430*, 128459. 10.1016/j.jhazmat.2022.128459.

45. Wang, S., Han, Q., Wei, Z., Wang, Y., Xie, J., and Chen, M. (2022). Polystyrene microplastics affect learning and memory in mice by inducing oxidative stress and decreasing the level of acetylcholine. Food Chem Toxicol *162*, 112904. 10.1016/j.fct.2022.112904.

46. Liu, X., Yang, H., Yan, X., Xu, S., Fan, Y., Xu, H., Ma, Y., Hou, W., Javed, R., and Zhang, Y. (2022). Co-exposure of polystyrene microplastics and iron aggravates cognitive decline in aging mice via ferroptosis induction. Ecotoxicol Environ Saf *233*, 113342. 10.1016/j.ecoenv.2022.113342.

47. Liu, X., Zhao, Y., Dou, J., Hou, Q., Cheng, J., and Jiang, X. (2022). Bioeffects of Inhaled Nanoplastics on Neurons and Alteration of Animal Behaviors through Deposition in the Brain. Nano Lett *22*, 1091-1099. 10.1021/acs.nanolett.1c04184.

48. Kwon, W., Kim, D., Kim, H.Y., Jeong, S.W., Lee, S.G., Kim, H.C., Lee, Y.J., Kwon, M.K., Hwang, J.S., Han, J.E., et al. (2022). Microglial phagocytosis of polystyrene microplastics results in immune alteration and apoptosis in vitro and in vivo. Sci Total Environ *807*, 150817. 10.1016/j.scitotenv.2021.150817.

49. Jeong, B., Baek, J.Y., Koo, J., Park, S., Ryu, Y.K., Kim, K.S., Zhang, S., Chung, C., Dogan, R., Choi, H.S., et al. (2022). Maternal exposure to polystyrene nanoplastics causes brain abnormalities in progeny. J Hazard Mater *426*, 127815. 10.1016/j.jhazmat.2021.127815.

50. Estrela, F.N., Guimaraes, A.T.B., Araujo, A., Silva, F.G., Luz, T.M.D., Silva, A.M., Pereira, P.S., and Malafaia, G. (2021). Toxicity of polystyrene nanoplastics and zinc oxide to mice. Chemosphere *271*, 129476. 10.1016/j.chemosphere.2020.129476.

51. Xu, D., Ma, Y., Han, X., and Chen, Y. (2021). Systematic toxicity evaluation of polystyrene nanoplastics on mice and molecular mechanism investigation about their internalization into Caco-2 cells. J Hazard Mater *417*, 126092. 10.1016/j.jhazmat.2021.126092.

52. Deng, Y., Zhang, Y., Lemos, B., and Ren, H. (2017). Tissue accumulation of microplastics in mice and biomarker responses suggest widespread health risks of exposure. Sci Rep *7*, 46687. 10.1038/srep46687.
